# A Storage Management System with Supercapacitors for Piezo–Thermoelectric Energy Harvesting Devices

**DOI:** 10.3390/mi17060723

**Published:** 2026-06-15

**Authors:** George-Claudiu Zărnescu, Lucian Pîslaru-Dănescu, Marius Popa, Ioan Stamatin

**Affiliations:** 1 Laboratory of Sensors/Actuators and Energy Harvesting, National Institute for Research and Development in Electrical Engineering ICPE-CA, 030138 Bucharest, Romania; george.zarnescu@icpe-ca.ro; 2 Laboratory of Microprocessing and Rapid Prototyping, Department of Electromechanical Systems and Technologies, National Institute for Research and Development in Electrical Engineering ICPE-CA, 030138 Bucharest, Romania; marius.popa@icpe-ca.ro; 3 3Nano-SAE Research Center, Faculty of Physics, University of Bucharest, 077125 Magurele, Romania; istarom@3nanosae.org

**Keywords:** piezoelectric, thermoelectric, photovoltaic, average summing circuit, automatic voltage regulator, pulse width modulation (PWM), ultra-low power

## Abstract

Two semiflexible piezoelectric composite plate structures were developed, incorporating 1 × 9 and 2 × 9 arrays of PZT elements mounted on brass discs and mechanically secured by pop rivets within a thin plastic foil spacer positioned between two copper-clad PCB layers. This configuration provides reliable electrical contact, adequate mechanical compliance, and efficient conversion of mechanical vibration energy into electrical energy. In addition, a multifunctional thermoelectric device was realized, consisting of four cubic modules arranged around a rectangular tube and enabling both handheld operation and coupling to hot or cold surfaces. Each cube is equipped with optimized finned heat sinks and integrates four thermoelectric elements on each face. Experimental results show that each cube generates approximately 6 mW, when handheld and with icy water injected into the central tube, demonstrating its suitability as a compact and versatile thermal energy harvester. Under low-light conditions, a solar panel is supplemented by this hybrid piezoelectric–thermoelectric energy harvesting system that combines the output of a piezoelectric composite plate with the dual outputs of a thermoelectric device using an electronically isolated summing block to ensure source decoupling. Energy storage and management are implemented using a capacitor buffer for the piezoelectric device, two voltage boosters for the thermoelectric outputs, and an automatic ultra-low-power pulse width modulation buck regulator for charging supercapacitors at 5 V.

## 1. Introduction

The growing demand for sustainable, autonomous, and self-powered electronic systems has accelerated research into energy harvesting technologies that capture ambient mechanical, thermal, and environmental energy. These systems aim to supply power to low-power devices such as wireless sensor nodes, Internet of Things (IoT) devices, wearable electronics, and biomedical monitoring systems, reducing dependence on conventional batteries and enabling longer operational lifetimes. Among the various energy sources, mechanical vibrations, body heat, and environmental thermal gradients have emerged as particularly promising for portable and wearable applications.

Flexible piezoelectric devices, especially those fabricated with sputtered thin films or low-temperature 0–3 composite thick films, have been widely explored for converting mechanical stress into electrical energy. Sputtered thin films, including materials such as AlN, ZnO, and PZT deposited on flexible substrates such as Kapton, provide precise control over crystal structure and film composition, enabling lightweight, low-cost, and high-performance energy harvesters [1]. Low-temperature composite films, such as PZT-based PiezoPaint, offer piezoelectric coefficients of 35–43 pC/N, broadband acoustic response, and mechanical flexibility, allowing integration into curved or stretchable structures for biomedical, acousto-optic, and wearable sensing applications [2,3]. Enhancements such as bilaminated composite vibrators, 2-2 or 1-3 composite structures, and screen-printed P(VDF-TrFE)/BaTiO_3_ films have further improved displacement, lowered resonance frequencies, and increased output power, highlighting the potential of flexible piezoelectric devices in energy harvesting systems [4,5,6].

Parallel to piezoelectric systems, thermoelectric generators (TEGs) have gained attention as a means to harvest energy from temperature differences, including the human body. Recent studies have demonstrated flexible, wearable TEGs embedded into clothing or sportswear, such as wrist guards, headbands, leg guards, and tights, where thermal gradients between skin and the environment are converted into electrical energy [7,8,9].

The performance of both piezoelectric and thermoelectric energy harvesters is strongly influenced by device geometry, material properties, and environmental conditions. For piezoelectric harvesters, factors such as initial free-end levitation position, laminate structure, and polymer matrix composition affect resonance frequency, displacement, and output power [4,5]. For TEGs, heat transfer efficiency, thermal resistance matching, and the design of heat sinks are critical for maximizing the internal temperature gradient and power generation [10]. Recent innovations include copper-foam heat sinks, lateral planar structures, and flexible, conformable TEG arrays integrated into clothing to maintain comfort and fashionability while harvesting energy from the human body [9,10]. Similarly, small-scale TEGs embedded into devices like wireless earphones or 3D-printed shoe soles illustrate the potential of thermoelectric energy harvesting in consumer electronics and wearable technologies, demonstrating measurable voltage and power outputs even under small temperature gradients [7,11,12].

Experimental results show that single TEG modules can produce up to 250 μW at modest temperature differences, while multi-module integration can reach nearly 1 mW. Integrating flexible supercapacitors as energy storage elements ensures stable power output and seamless interaction with wearable devices, providing clean and persistent energy for low-power sensors [13]. Innovative TEG designs, such as Cu–Ni and Cu–Co thick-film devices on Cu-clad polyimide substrates, leverage lateral heat-flow structures and optimized thermo-leg geometries to enhance Seebeck coefficients and power densities, achieving significant improvements over traditional metal-based TEGs [12].

To effectively utilize the energy captured from multiple sources, advanced energy management systems are required. Supercapacitors offer high energy density, rapid charge–discharge capability, and compatibility with miniaturized electronics, making them suitable for hybrid storage solutions in wearable and IoT devices. Coupling supercapacitors with power-management circuits, such as low-voltage DC–DC boost converters and multi-input synchronous electronic charge extraction (EMI-SECE) circuits, allows energy harvested from piezoelectric, thermoelectric, and photovoltaic transducers to be efficiently regulated, stored, and delivered to low-power devices [14,15,16,17,18]. EMI-SECE circuits, for example, enable simultaneous energy extraction from multiple transducers with varying phase differences, achieving high conversion efficiencies and adaptability to asynchronous vibration sources. Similarly, thermoelectric DC–DC boost converters have demonstrated self-startup capabilities at input voltages as low as 10–70 mV, using adaptive maximum power point tracking (MPPT) and zero-current switching techniques to optimize energy harvesting under ultra-low-voltage conditions [15,16,17,18]. These developments highlight the integration of energy harvesting, power conversion, and energy storage as a unified approach to powering autonomous devices.

Overall, the convergence of flexible piezoelectric devices, wearable TEGs, and advanced energy management systems is enabling a new generation of self-powered electronics. By combining photovoltaic, mechanical, thermal, and hybrid energy harvesting with compact and efficient storage solutions, researchers are creating practical systems capable of powering wearable sensors, biomedical devices, and low-power IoT applications without reliance on conventional batteries. Ongoing challenges include improving material performance, optimizing geometries, and developing scalable fabrication methods while maintaining user comfort and device reliability. These advances promise to expand the scope of energy-autonomous electronics across healthcare, wearable technology, and smart infrastructure domains.

This paper is organized as follows. First, in Section 2.1 and Section 2.2, the design and experimental setup of the piezoelectric and thermoelectric harvesting devices are presented, with emphasis on the structural configuration, electrical interconnection, and measurement methodology adopted for both systems. Next, Section 3.1 provides the theoretical and experimental investigation of the piezoelectric device, highlighting the influence of strain distribution, element positioning, applied pulling force, and array configuration on the generated voltage, current, and harvested power. This chapter brings a detailed correlation between the mechanical deformation of the composite plate and the electrical response of the piezoelectric elements, together with a comparative assessment of the nine-element and 18-element structures. Section 3.2 then examines the thermoelectric device from both theoretical and experimental perspectives, focusing on heat-transfer mechanisms, temperature-gradient evolution, internal resistance behavior, and power generation under different cooling and heating conditions. This chapter contributes a system-level interpretation of thermoelectric performance by linking the measured electrical output to the thermal behavior of the proposed structure. Overall, the paper brings a combined analysis of two complementary energy-harvesting approaches and provides experimental evidence for their suitability in low-power autonomous applications.

The final Section 3.3 describes the electronic energy management and storage circuits. The photovoltaic, piezoelectric, and thermoelectric sources are conditioned to a specified voltage range and subsequently combined while maintaining electrical isolation using an averaging summing circuit. The piezoelectric composite plate employs a polyester buffer capacitor (10–20 µF, 50 V rating) to filter and reduce the output voltage. The thermoelectric device is interfaced with two Meissner oscillators specifically designed for low-power operation, providing a high voltage gain of approximately 48× and low-voltage start-up (below 100 mV). Following the summing stage, an automated ultra-low-power buck regulator is used to charge the supercapacitors.

This work introduces several key innovations. In contrast to conventional in-house fabricated flexible piezoelectric harvesters [3,5], the proposed composite structure provides a cost-effective and readily implementable solution based on an array of mechanically pressed PZT–brass elements and embedded PCB interconnections patterned on both sides of thin PCB laminates. Each piezoelectric element is individually connected to a SMD full-wave Schottky rectifier, enabling independent charge extraction and allowing the array to function collectively as an equivalent larger piezoelectric disc. This architecture also mitigates charge cancellation effects between adjacent piezoelectric elements. Electrical contact is achieved by gentle mechanical compression using pop rivets, while plastic spacers with a thickness close to the combined PZT–brass element thickness prevent excessive clamping, pre-stressing, or mechanical damage.

In parallel, the multifunctional thermoelectric device based on cube-shaped finned heat-sink assemblies integrated around a square aluminum tube was developed to support both handheld operation and fluid-assisted thermal-gradient generation. The proposed innovation resides in the use of the central square aluminum tube as a multifunctional structural and thermal interface element. Furthermore, metal additive manufacturing enables monolithic integration of the cube-shaped finned heat sink with the square aluminum tube, facilitating a compact design and improved thermal coupling.

Combining electrical sources with substantially different output characteristics through conventional series or parallel connections remains challenging. This paper presents an electrically isolated averaging and summing circuit that enables the integration of asynchronous outputs from piezoelectric, thermoelectric, and photovoltaic transducers while preserving source isolation.

To enable efficient low-voltage operation, the thermoelectric outputs are conditioned using Meissner-based ultra-low-voltage oscillator circuits, followed by an automated ultra-low-power PWM buck stage for controlled supercapacitor charging. The proposed system therefore demonstrates a compact and scalable approach for powering autonomous low-power electronics through simultaneous harvesting of mechanical, thermal, and ambient light energy.

## 2. Materials and Methods

### 2.1. Design and Experimental Setup of the Piezoelectric Device

**Figure 1:**
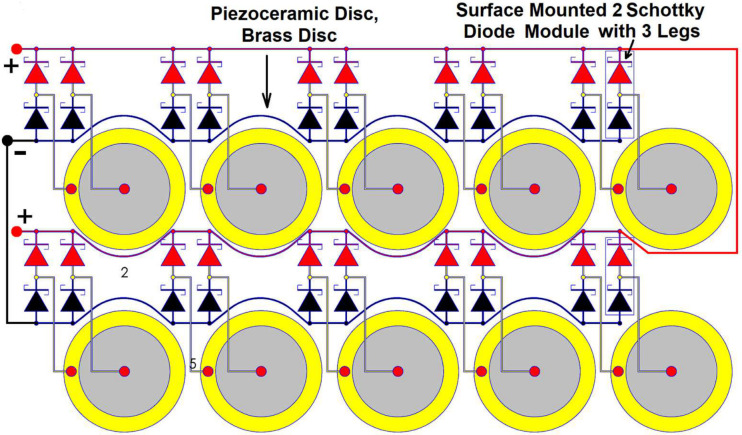
Electrical schematic of the 210 mm long composite PZT plate.

For round piezoelectric elements made of lead zirconate titanate (PZT) with a diameter of 15 mm and mounted on 20 mm brass disks, a semi-flexible composite matrix structure was adopted. Figure 1 presents the general electrical configuration of the system. For clarity, only five PZT elements arranged in a single row are illustrated; however, the electrical connections for configurations containing 9 or 18 elements follow the same principle.

**Figure 2:**
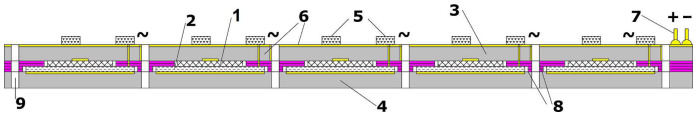
Lateral view of the piezoelectric composite plate.

**Figure 3:**
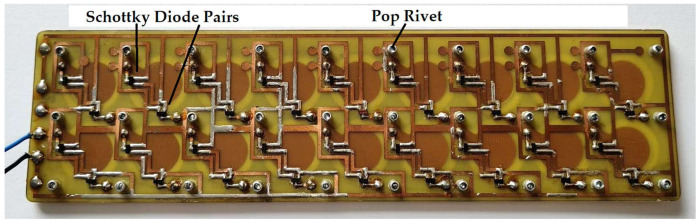
Composite plate (210 mm × 56 mm) encapsulating a 2 × 9 array of piezoceramic discs bonded to brass substrates, each with a diameter of 20 mm.

The elongated composite plate utilizes both the piezoelectric and pyroelectric effects within the same material system. The structure consists of piezoceramic elements (1) bonded to brass disks (2), and copper traces on the printed circuit boards (PCBs), together with pop rivets, ensure both electrical and mechanical contact (see Figure 2 and Figure 3). The brass disks and circular copper pads of varying diameters on the PCB traces function as vibration amplifiers and contribute to the establishment of a temperature gradient across the structure.

The assembly is composed of two printed circuit boards, each with a thickness of 0.4 mm, which are joined using small pop rivets and plastic spacers. The spacers have the same thickness as the piezoceramic elements (0.6 mm) to prevent mechanical damage or pre-stressing of the PZT elements. These plastic spacers contain circular openings with diameters of 21 mm and 16 mm, into which the piezoceramic elements are inserted.

Each PCB is fabricated as a double-layer board. On the inner surfaces, circular copper contact pads are printed using an ultraviolet (UV) flatbed printing process, after which the outer copper layer is chemically etched. The circular copper contacts are directly connected to the piezoceramic elements by mechanically pressing the two PCB plates together.

The boards are designed with a length at least five times greater than their width. On the bottom side, circular copper contacts are provided for the piezoelectric elements, as well as electrical interconnections (6) leading to the outer surface where surface-mounted rectifier modules (5) are installed. Each rectifier module incorporates a BAS40-04 Schottky diode array comprising two Schottky diodes connected in series. Two such modules form a full rectifier bridge, and all rectifier bridges are connected in parallel in order to increase the total rectified current.

Both the plastic spacer plate and the PCB layers include four fixing holes arranged around each piezoelectric disk. These holes secure the copper contacts and prevent lateral movement of the disks between the contact pads. The main disadvantage of the composite board is associated with the fastening system, which introduces additional structural rigidity (see element 5). Nevertheless, the electrical connector pad (6) linking the upper and lower layers cannot be eliminated in the current configuration. A practical solution to maintain the design while preserving flexibility is to employ thinner PCB substrates (0.2–0.4 mm), smaller pop rivets with diameters below 2.4 mm, and longer PCB plates.

Since each piezoelectric element is connected to an individual full-wave Schottky bridge rectifier, the electrical charge generated by each element is transferred independently to the common storage capacitor. Consequently, the total accumulated charge represents the sum of the charges produced by all active piezoelectric elements.

The bending moment and force distribution along the composite plate are proportional to the local mechanical strain. Consequently, the electrical charge generated by each piezoelectric element depends on its spatial position along the plate. The pulsed pulling force was applied using two Pesola Medio-Line 40025 Precision Scale, Pesola AG, Schindellegi, Switzerland (25 N capacity) and Pesola Medio-Line 40050 Precision Scale, Pesola AG, Schindellegi, Switzerland (50 N capacity). The first scale has a resolution of 0.2 N per gradation, while the second provides a resolution of 0.5 N per gradation. The applied forces were further verified using a portable electronic scale with a measurement precision of 5 g.

The maximum bending deformation occurred in the central region of the plate; consequently, the piezoelectric elements located in this region generated the highest current and voltage outputs. The measurement setup consisted of two non-elastic copper wires fixed at the edges of the composite plate, near the pop rivets, in order to prevent lateral displacement. The wires were also connected on the upper side of the plate at the midpoint to enable the application of the pulling force.

**Figure 4:**
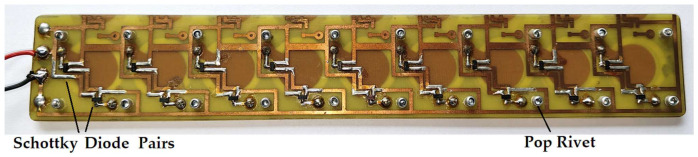
Composite plate (210 mm length, 32 mm width) encapsulating an array of nine piezoceramic–brass discs elements, each with a diameter of 20 mm.

The same mechanical configuration was used for both composite plates: the one containing nine piezoelectric elements and the one containing eighteen piezoelectric elements (see Figure 3 and Figure 4). Both composite plates were clamped at the center using a portable pneumatic vice. Although different clamping methods have been reported, the overall structural concept of the harvesting devices remains similar. In earlier studies, composite plates were clamped at the edges and energy was harvested under central impact pressure pulses [19,20], yielding a strong response for lead-based piezoceramic–polymer composites, with a power density in the range of 3165–8165 μW/cm^3^.

By contrast, in the present work, the composite plates were designed so that the impact force was applied at both ends. A similar mechanical configuration has been reported by Truong and Roundy [21], who investigated a center-clamped piezoelectric receiver with magnetic tip masses, demonstrating the feasibility of symmetric end excitation in a centrally fixed piezoelectric structure. By employing symmetric end excitation with magnets, the composite plate could be integrated into a hybrid piezoelectric–electromagnetic energy harvesting device for applications such as footstep-powered tiles. Although the geometry differs, a similar fixation concept has been used in other harvesting devices, where circular piezoelectric plates deposited on metallic substrates were clamped at the center [22].

**Figure 5:**
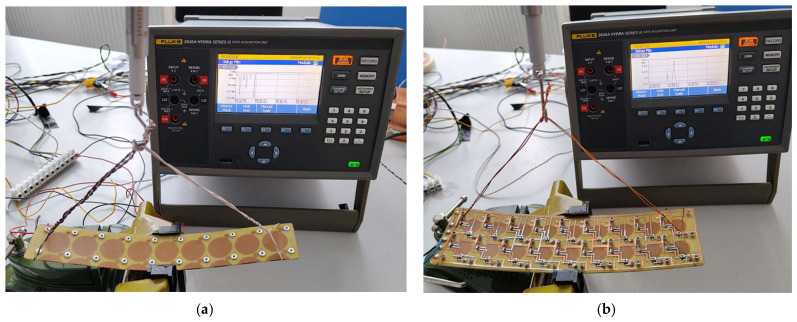
(**a**) Current and voltage measurements for the composite plate with 9 piezoelectric elements; (**b**) current and voltage measurements for the composite plate with 18 piezoelectric elements.

Using this identical mechanical setup, different pulling forces ranging from 5 N to 50 N were applied manually within the shortest possible time interval. Experimental measurements indicated an effective excitation frequency in the range of approximately 3–5 Hz. This range is lower than the 6–10 Hz frequencies reported for certain human arm movement and vibration phenomena in the literature [23,24]. The resulting current and voltage were measured, as shown in Figure 5a,b.

Two channels of the Fluke 2638A Hydra Series III Data Acquisition Unit, Fluke Corporation, Everett, Washington, USA, were configured for transient voltage and current measurements. Channel 122 was used to measure the current output of each composite plate, while Channel 107 was configured to measure the DC voltage output of the composite plates containing nine and eighteen piezoceramic elements. Channels 121 and 122 are specifically designed for current measurements, as they incorporate internal shunt resistors for this purpose. Accordingly, Channels 121 and 122 were enabled for DC current measurements. The Mx + B scaling function was also activated to automatically scale and compute the measured current values.

The composite plate containing nine piezoelectric elements requires a lower bending force to generate usable electrical power (Figure 5a). The effective force interval for this configuration ranges between 10 N and 40 N. In contrast, the composite plate containing eighteen piezoelectric elements operates within a higher force interval, between 15 N and 50 N (Figure 5b). This difference arises primarily from the plate width-to-length ratios (56/210 and 32/210), as well as from the increased stiffness of the plate that is present in the second configuration.

For the composite plates containing nine and eighteen piezoceramic elements, additional rms measurements were performed using the Agilent 34461A Digital Multimeter, Keysight Technologies, Santa Rosa, CA, USA, to verify the generated current and voltage values. While the Fluke 2638A Hydra Series III Data Acquisition Unit provides graphical visualization of the voltage and current transient behavior over the entire measurement period, the Agilent 34461A Digital Multimeter displays only the effective (steady or rms) values of the measured quantities. In order to verify and analyze in detail the transient bending and elastic recovery phenomena, the voltage signals were acquired using a Tektronix TDS2014B digital oscilloscope, Tektronix, Inc., Beaverton, OR, USA. The oscilloscope time base was set to approximately 500 ms/div (5 s observation window), allowing multiple transient voltage pulses associated with manual excitation to be captured within a single acquisition.

### 2.2. Dimensioning and Experimental Setup of the Thermoelectric Device

**Figure 6:**
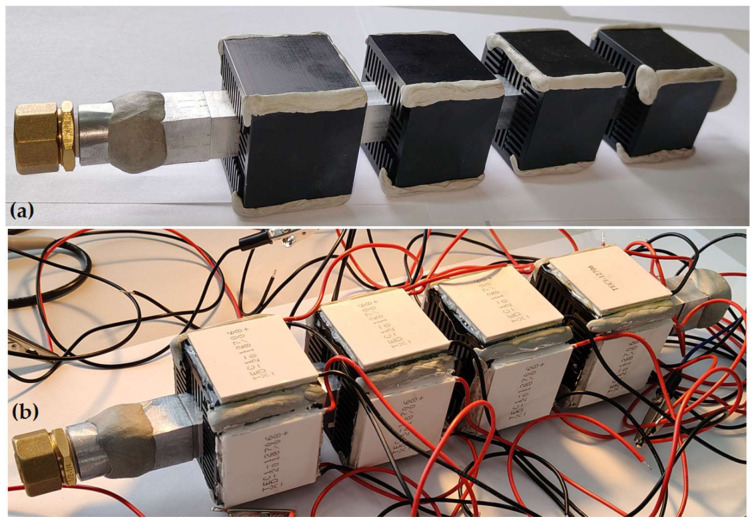
Manufacturing process of the thermoelectric device: (**a**) positioning and permanently fixing the cube heat sinks onto the aluminum square tube using epoxy putty; (**b**) positioning and permanently bonding (on sides) the thermoelectric module plates onto the cube heat sinks using epoxy adhesive.

The thermoelectric conversion device was constructed using four cube-type thermoelectric modules mounted on a square aluminum tube with a wall thickness of 3 mm. In the region designated for mounting the aluminum heat sink assemblies, the external dimensions of the 20 × 20 mm square, 350 mm long, aluminum tube were reduced by precision milling to 18.6 × 18.6 mm (see Figure 6a,b).

Specifically, 0.7 mm of material was removed from each face over a length of 250 mm, resulting in a reduced cross-sectional dimension of 18.6 × 18.6 mm along this section.

Each thermoelectric module was built from four identical aluminum heat sinks assembled into a cubic configuration. The heat sinks (manufactured by Fischer Elektronik GmbH & Co. KG, Lüdenscheid, Germany) have a square base of 50.8 × 50.8 mm, a base thickness of 4 mm, and fins with a length of 12.5 mm, resulting in a total height of 16.5 mm. Each heat sink contains 225 fins of 1.5 mm thickness, with an inter-fin spacing of approximately 1.8 mm. This spacing allows the mechanical interlocking of four identical heat sinks into a cube geometry. Following assembly, the heat sinks were bonded along their edges using an epoxy putty to ensure structural integrity.

A minimum spacing of 20 mm was maintained between adjacent aluminum cubes to allow subsequent longitudinal adjustment along the aluminum tube. This adjustment enabled the insertion of thermally conductive interface films with a thickness of 0.1–0.2 mm, thereby improving thermal contact between the aluminum tube and the heat sink fins.

After mechanical stabilization of the aluminum cubes, thermoelectric elements of type TEC1-12706 (40 × 40 mm cross-section, approximately 4 mm thickness) were bonded to the outer surfaces using a two-component epoxy adhesive applied along the edges.

A copper sheet with a length of 250 mm and a thickness of 1 mm was progressively rolled onto a 60 × 60 mm square steel tube with a wall thickness of 2 mm, forming four planar faces and achieving final edge alignment at one corner. This formed the external heat distribution layer of the assembly.

Electrical connections were then completed. The four thermoelectric elements mounted on each cube were connected in series in order to increase the output voltage. The copper sheet was expected to promote thermal homogenization of the heat source across all faces of the cube, albeit with a reduced temperature gradient. The two output conductors from each module were routed through the remaining corner spaces of the assembly, after all thermoelectric modules were pushed inside the copper tube.

To ensure mechanical rigidity and optimal thermal contact between the copper sheet and the outer ceramic surfaces of the thermoelectric elements, multiple clamping brackets (at least two per module, ten in total) were applied. The clamping process continued until all visible gaps between the ceramic plates and the copper sheet were eliminated.

For sealing and mechanical reinforcement, a polyurethane-based adhesive was initially injected at one end of each corner, sealing the edges between the heat sinks and the ceramic plates of the thermoelectric elements and securing the copper sheet in position. Subsequently, a two-component epoxy resin was poured sequentially into each corner (the assembly was slightly inclined during pouring and allowed to cure in a horizontal position). Upon curing, the epoxy resin provided permanent bonding between the copper sheet and the edges of both the heat sinks and the ceramic plates. This procedure was repeated until all four corners were fully sealed, and the entire structure was rigidized.

Prior to final sealing, the fins of each heat sink were coated with a thermally conductive compound to enhance heat transfer efficiency. The remaining cavities of the assembled heat sink cube may also contribute to improved thermal insulation between the hot and cold sides. If both sides are completely thermally insulated and properly sealed, these cavities could potentially maintain a vacuum or phase-change material powder rather than air, thereby further reducing heat transfer between the two sides [25,26].

The hot-side temperature of the thermoelectric device was measured at two locations, on the left and right sides of the outer copper sheet layer. The cold-side temperature was measured on the aluminum tube through which cold or ice-cold water was injected.

For this measurement, the sealed rear side of the aluminum tube was selected, as the opposite side contains a copper inlet with a fillet used to introduce the water and to thread the cap onto the copper–aluminum round pipe adapter. This sealed configuration was adopted to resemble the structure of a vacuum bottle, with the purpose of maintaining the injected water at a low temperature inside the tube for as long as possible.

**Figure 7:**
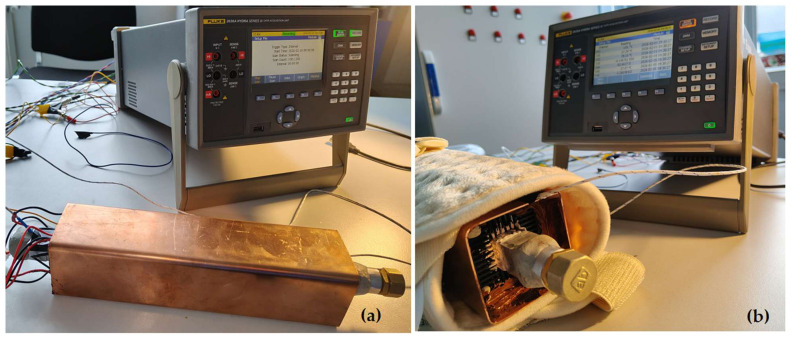
Time-dependent temperature, voltage, and current measurements of the thermoelectric device: (**a**) operating solely with cold or ice-cold water; (**b**) filled with cold water, with the outer copper sheet covered by a heating blanket.

For the measurements shown in Figure 7a, a Fluke 2638A Hydra Series III Data Acquisition Unit was used to record voltage, current, and temperature data. Four channels were allocated for electrical measurements: Channels 103 and 107 for DC voltage and Channels 121 and 122 for automatic current measurements, using internal shunt resistors. In addition, Channels 101, 102, and 105 were configured for K-type thermocouple measurements and were used to measure temperatures at the cold and hot sides of the device. One thermocouple was fixed with tape at one end of the 20 × 20 mm aluminum tube, near the back seal, to monitor the cold-side temperature. The second thermocouple was attached with insulating tape to the outer copper sheet on the hot side, where the first and second cube-type thermoelectric modules were located inside the device, as illustrated in Figure 7b.

The third K-type thermocouple, connected to Channel 105, was used to monitor the temperature on the opposite side of the copper sheet on the hot side, where the third and fourth cube-type thermoelectric modules were positioned along the tube.

The results, discussed in the following chapters, indicate that static water cooling lowered the cold-side temperature of the thermoelectric device by about 3 °C, but only for a duration of several minutes. To sustain the temperature gradient over a longer period, forced water cooling or the incorporation of phase-change materials inside the aluminum tube is recommended.

The present design may be further modified by retaining only two neighboring thermoelectric modules above the cube-type heat sinks and by using solar thermal energy through a solar concentrator together with dynamically flowing cold water [27]. In addition, the module in its current configuration may also be used during winter, provided that the end insulation is improved by partial embedding in the ground, thus exploiting the temperature gradient between the soil and the ambient cold air [28].

## 3. Results

### 3.1. Theoretical and Experimental Investigation of the Piezoelectric Device

The electric charge generated by a piezoelectric element is directly proportional to the local mechanical strain, which in turn is proportional to the bending moment at the element’s position along the composite plate. Therefore, the electrical contribution of each element depends on its spatial location relative to the region of maximum curvature.

Because the maximum bending strain and curvature occur near the clamped central region of the plate, the piezoelectric elements located closest to the center generate the highest voltages and currents.

As the distance from the center increases, the bending moment and corresponding strain decrease progressively, leading to reduced electrical output from elements positioned toward the plate edges.

Due to the independent rectification stage assigned to each piezoelectric element, capacitive voltage equalization between elements is avoided. Instead, the rectified charges are cumulatively transferred to the storage capacitor. As a result, the output voltage depends on the total accumulated charge, pulse duration, and hand-pulling time, while the output current corresponds to the sum of the individual rectified currents.

For the investigated configurations (1 × 9 and 2 × 9 piezo elements), the experimentally validated proportionality coefficients describing the spatial strain distribution along one half of the plate are described by a=0.99 and a=1.16.

(1) $$k\left(x\right)=1-a\cdot {\left(2x/L\right)}^{2}$$

Distance x from the composite plate center gradually changes from 22 mm up to 88 mm, with a step size of 22 mm. The distance between pop rivets centers is also 22 mm. Each piezo is surrounded by four pop rivets.

For a=0.99, $k=\left(1.00,\;0.96,\;0.80,\;0.60,\;0.30\right)$ and for a=1.16, $k=\left(1.00,\;0.95,\;0.79,\;0.54,\;0.18\right)$. The coefficients k were experimentally determined from at least 20 repeated measurements based on the mean voltage and current values obtained at each applied force. These measurements were conducted with only the central SMD Schottky rectifier bridges connected, after which the remaining rectifiers were progressively added to the piezoelectric-element columns, and the measurements were repeated.

Across both plates (nine and 18 piezoelectric elements), the quadratic fitting model showed strong agreement with the experimental data, with coefficients of determination (R^2^) values ranging from 0.990 to 0.998, correlation coefficients from 0.993 to 0.998, and average relative errors between approximately 2% and 5% for the measured voltage.

These coefficients represent the relative contribution of the piezoelectric elements to the strain distribution along the plate.

For an ideal symmetric bending configuration, the strain distribution is expected to vary approximately quadratically (decrease) with the distance from the clamped center. This ideal case corresponds to a=1, giving k=(1.00,0.96,0.82,0.60,0.30) at positions $x=\left(0,22,44,66,88\right)\;mm$ from the plate center. The experimentally obtained values for a=0.99 and a=1.16 are close to this ideal distribution, indicating that the proposed geometry closely matches the mechanical strain profile of the bending plate. Therefore, the geometry can be considered near-optimal for coupling the strain field to the spatial distribution of the piezoelectric elements.

For short, when the plate is clamped at the center and loaded at the edges, the bending moment—and therefore the mechanical strain—is maximum at the center and decreases gradually toward the edges, following an approximately quadratic law. Because the piezoelectric voltage and generated charge are proportional to the local strain, the elements located near the center produce the largest electrical output, while those toward the edges generate progressively smaller voltages and currents.

By spacing the piezoelectric discs uniformly (22 mm center-to-center) along the plate length, the array samples the strain field at regular intervals. This configuration ensures that the central elements operate near their maximum strain while the outer elements still contribute useful electrical charge instead of occupying regions where the deformation would be too small to produce significant output. Furthermore, the symmetric placement of two piezoelectric discs across the plate width at each longitudinal position allows both sides of the plate to experience nearly identical deformation. This symmetry improves the effective current generation because the electrical contributions from elements subjected to similar strain are summed after rectification.

The combination of optimized inter-element spacing, symmetric arrangement, and individual Schottky rectification enables the piezoelectric array to capture a substantial portion of the bending strain energy available in the plate and to function collectively as an equivalent larger piezoelectric disc. Consequently, the system attains a relatively high electrical output (approximately 63 V and 5.2 mW) using only 18 small piezoelectric elements.

This distribution indicates that the entire assembly that contains nine piezoelectric elements behaves electrically as approximately 5.92–6.32 centrally located piezoelectric elements operating under maximum strain conditions. In addition, this distribution shows that the entire assembly incorporating 18 piezoelectric elements behaves electrically as approximately 11.84–12.64 centrally located piezoelectric elements operating under maximum strain conditions (at 47 N). The bending moment and the curvature distribution is not linear, but it fits very well to this quadratic model, from Equation (1).

Mechanically, the system operates in a clean and symmetric manner, featuring a centrally stiffened plate and thin PCB laminates (0.4 mm each), with the neutral axis reinforced by rivets. The rectifier-per-piezo configuration offers several advantages: it enables proper (non-destructive) charge summation, prevents capacitive cancellation, avoids losses due to phase mismatch, and maximizes the harvested energy by capturing additional contributions even from the smallest curvatures present at the plate edges.

(2) $$I_{ptot}=\frac{4}{\pi }I_{p}\sum_{i=0}^{4}n_{i}k_{i},\;where\;I_{p}=C_{p}U_{p}\omega =2\pi fC_{p}U_{p},\;U_{p}=F_{p}/A$$

In the center of the plate $n_{0}=2$, $k_{0}=1,$ and $n_{1\ldots 4}=4,$ four piezoelectric elements are distributed symmetrically along the plate, for 2 × 9 piezoelectric elements. For the plate with nine piezoelectric elements $n_{0}=1$, $k_{0}=1,$ and $n_{1\ldots 4}=2$, one is distributed symmetrically to the left side, and the other is distributed symmetrically to the right side (from the center). The electromechanical coupling factor A~3 N/V was determined experimentally for the plate with nine piezoelectric elements. In addition, for the composite plate containing 18 piezoelectric elements, the electromechanical coupling factor was A~4.1 N/V. Theoretically, this factor depends on the effective stiffness, Young modulus, composite disk geometry (thickness and surface area), and piezoelectric coefficients.

The electromechanical coupling factor for the composite plate containing nine piezoelectric elements was experimentally determined to be approximately A~3 N/V. For the composite plate containing 18 piezoelectric elements, the coupling factor increased to approximately A~4.1 N/V. Theoretically, this factor depends on the effective stiffness, Young’s modulus, geometric parameters of the composite disk (including thickness and surface area), and the piezoelectric coefficients of the active material. Although the composite plate containing 18 piezoelectric elements exhibited a larger electromechanical coupling factor, the softer composite plate can generate higher output voltages under mechanical excitation due to its lower effective stiffness. The reduced stiffness allows larger bending deformation and, consequently, higher mechanical strain in the piezoelectric layer under the same applied force. Since the generated electrical charge is directly related to the induced strain, the more compliant structure can produce higher open-circuit voltages despite its lower force factor. In contrast, the stiffer composite plate undergoes smaller deformation under identical loading conditions, resulting in reduced strain-induced voltage generation. All voltage measurements were performed under open-circuit conditions.

Since the electrical capacitance of a single piezoceramic element with a diameter of 15 mm, bonded to a 20 mm brass disk, is $C_{p}=11\;nF$, and the maximum hand-motion frequency is approximately 4–5 Hz, the average current generated by one central piezoelectric element after full-bridge rectification was estimated to be $I_{p}\approx 6-7\;\mu A$. This value is in good agreement with the experimentally measured current obtained under a bending force of 47 N for the composite plate containing 18 piezoelectric elements.

For the same bending force, the voltage generated by the central piezoelectric element mounted in the composite plate containing the 1 × 9 piezoelectric array was $U_{p}=15\;V$, whereas the corresponding voltage measured for the composite plate containing the 2 × 9 piezoelectric array was $U_{p}=11\;V$. The higher voltage generated by the 1 × 9 configuration is attributed to its lower effective stiffness, which allows larger bending deformation and consequently higher strain levels in the piezoelectric material.

As said earlier, the individual (per column) voltage measurements were performed before mounting the remaining SMD Schottky rectifier bridges on the composite plate surface; only the two central rectifier bridges were connected during the initial measurements. Subsequently, the Schottky rectifiers were progressively added for each column containing one or two piezoelectric elements, and the corresponding voltage measurements were repeated. Using these experimental data, the a and k coefficients were determined for each position on the composite plate.

**Figure 8:**
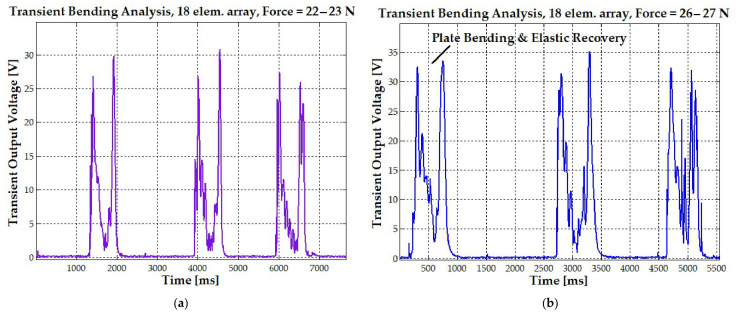
Transient output voltage of the plate containing an array of 18 piezoelectric elements during bending and elastic recovery: (**a**) at a pulling force of 22–23 N; (**b**) at a pulling force of 26–27 N.

The transient voltage response of the piezoelectric generator was investigated under different manually applied pulling forces. An increase in the output voltage was observed with increasing excitation force, and these measurements were in agreement with the mean voltage values reported for the composite plate containing 18 piezoelectric elements. Voltage waveforms were recorded using a Tektronix TDS2014B oscilloscope to examine the bending and elastic recovery behavior of the plate and to determine its actuation frequency. Each acquisition captured several voltage pulses generated during repeated manual excitations at a given force level. For an applied force of approximately 22 N, the pulse amplitudes ranged from 26 V to 31 V (Figure 8a). When the force increased to approximately 26–27 N, the pulse amplitudes increased to 31–35 V, while the corresponding actuation frequency was between 2.8 Hz and 4 Hz (Figure 8b).

**Figure 9:**
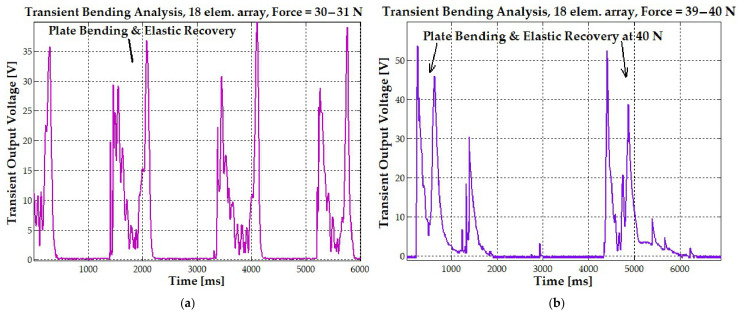
Transient output voltage of the plate containing an array of 18 piezoelectric elements during bending and elastic recovery: (**a**) at a pulling force of 30–31 N; (**b**) at a pulling force of 39–40 N.

For higher bending forces in the range of 30–40 N, the voltage amplitude increased from approximately 35–40 V to 46–52 V. This trend further confirms the proportional relationship between the output voltage and the total charge generated by the piezoceramic elements. In addition, charge generation occurred during both the bending and elastic recovery stages of the plate. As a result, the charge produced during each deformation cycle passed through the rectifier bridges in two separate events, producing two positive current and voltage peaks, as illustrated in Figure 9a,b.

For the plate incorporating 18 piezoelectric element array, an applied force of approximately 22–23 N produced a mean output voltage of 27.7 V. The corresponding standard deviation was 2.1 V, giving a relative standard deviation of 7.4%. At 26–27 N, the output voltage ranged between 31 V and 35 V. The mean value was 32.8 V. The standard deviation was 1.44 V, corresponding to a relative standard deviation of 4.4%. At 30–31 N, the maximum measured output voltage reached a mean value of 38.2 V. The standard deviation was 1.75 V, corresponding to 4.6% relative standard deviation.

For the nine piezoelectric element plate, at 29 N the mean output voltage was 22 V. The standard deviation was 0.8 V, corresponding to a relative standard deviation of 3.6%. At 36–37 N, the mean voltage increased to 26.5 V. The standard deviation was 0.88 V, corresponding to a relative standard deviation of 3.5%.

**Figure 10:**
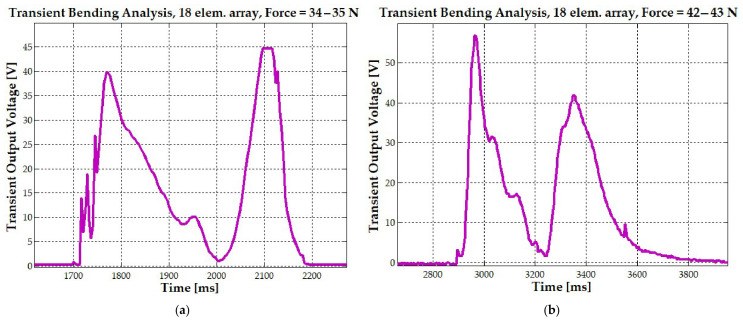
Transient output voltage for bending frequency identification: (**a**) at a pulling force of 34–35 N; (**b**) at a pulling force of 42–43 N.

Figure 10a,b show that the bending actuation frequency remained within the range of 3.3–3.6 Hz, whereas the elastic recovery of the plate, corresponding to its return to the horizontal position, occurred within approximately 180 ms, equivalent to a frequency of about 5.5 Hz. The voltage pulses recorded by the oscilloscope are also in good agreement with the mean voltage values measured using the Fluke 2638A Hydra Series III Data Acquisition Unit, thereby validating the experimental results obtained from both measurement systems. The use of individual Schottky rectifiers allows each piezoelectric element to independently harvest energy generated during both the bending and elastic recovery phases. The rectified contributions are subsequently combined at the common DC bus, resulting in a voltage amplitude of approximately 45 V for applied forces in the range of 34–35 N and a maximum voltage amplitude of 58 V for applied forces of 42–43 N.

All results are based on mean values obtained from at least 20 repeated measurements per force level. In some cases, the number of repetitions exceeded this minimum. This was due to the need for precise synchronization between manually applied force pulses and voltage acquisition.

Manual plate bending introduced errors ranging from 3.5% to 7.4%. Forces in the range of 15–35 N could be applied relatively consistently for both plates, resulting in smaller errors 3.5–4.6%. However, when the applied force exceeded approximately 39 N, manual bending became increasingly difficult, resulting in reduced force-control accuracy. This limitation is associated with the larger displacements required from the neutral axis. In vibration-testing applications, displacement amplitudes of 50 mm and 75 mm correspond to the maximum continuous stroke (peak-to-peak displacement amplitude) of large-scale electrodynamic or mechanical shaker systems. Shakers operating within this displacement class typically provide maximum dynamic force capacities of approximately 35–50 kN. However, such force levels are far beyond the requirements of the plates investigated in this study, for which only comparatively small forces in the range of 15–50 N were applied. In addition, the tested plates would require a dedicated mechanical adaptation system for end clamping and bending, integrated with the shaker assembly.

Since the focus of this study was the assessment of the energy-harvesting capabilities of the piezoelectric plates, rather than the implementation of a complete harvesting system, a manual bending procedure was employed. This approach avoided the additional mechanical complexity associated with shaker-based excitation, whose force capacities substantially exceed the loading requirements of the tested plates. Nevertheless, the use of an electrodynamic shaker would enable precise control of both the excitation force and bending frequency.

The manual excitation frequency observed during hand actuation (approximately 3–5 Hz) corresponds to the lower range of voluntary cyclic human motion reported in biomechanics studies [23,24]. At these frequencies, the hand–device system approaches its mechanical compliance limit, allowing larger bending amplitudes with minimal effort.

During the transient bending event, the storage capacitor integrates all independent charge contributions on the final bus, resulting in a higher accumulated voltage for a short time after each rectifier bridge. The piezoelectric elements operate as charge sources whose generated charge is proportional to the mechanical strain. The rms voltage rise $U_{bus}$ is ultimately limited by the diode conduction condition $k_{i}U_{p}>U_{bus}+2U_{D}$ and the electrical load, which determine the steady operating point of the system.

(3) $$U_{bus}=8fR_{bus}C_{p}U_{p}\sum_{i=0}^{4}n_{i}k_{i}$$

The measured output bus resistance for the composite plate with 18 piezoelectric elements was $R_{bus}=750\;k\Omega$. According to Equation (3), the estimated root mean square (rms) bus voltage at the maximum manual plate-bending frequency of 5 Hz was 46 V. Under the same operating conditions, a peak voltage of 62 V was experimentally measured at an applied force of 47 N. Because the harvested signal consisted of a sequence of non-uniform voltage pulses generated by manual excitation, no fixed theoretical relationship between the rms and peak voltages was assumed, and the following ratio was considered an experimentally derived parameter.

For the composite plate with 9 piezoelectric elements, the measured output bus resistance was $R_{bus}=470\;k\Omega$. Based on Equation (3), the estimated bus voltage at the maximum manual plate-bending frequency of 5 Hz was 19 V. Under the highest dynamic bending conditions of 5 Hz and 41 N, the measured peak voltage reached 32 V.

From experimental measurements, an rms-to-peak voltage ratio ranging between 0.6 and 0.75 was assumed.

A composite plate containing 18 PZT elements can deliver a maximum output power of approximately 5.2 mW. The composite plate containing 18 piezo–brass disk elements could be further optimized by increasing its length to 250–260 mm and adopting a 20-element piezoelectric array. This modification would reduce the plate stiffness and decrease the coupling factor A, potentially improving the overall energy harvesting performance. For a configuration with nine piezoceramic elements, the structure exhibits optimal performance when the plate length does not exceed 210 mm (analyzed case), with each element having a diameter of 20 mm and being arranged in a single row. The maximum thickness of the composite plate is approximately 1.4 mm, excluding the height of the surface-mounted Schottky diodes and the pop rivets.

Under a pulsed mechanical load exceeding 45 N, the system generates a maximum rectified voltage of 62 VDC under dynamic conditions and a rectified current of 84 µA. Measurements were performed using a Fluke 2638A Hydra Series III Data Acquisition Unit, which records the full time evolution of the voltage and current signals, allowing graphical visualization of the generated pulses. For different applied mechanical loads, the corresponding linear fitting coefficients obtained from these measurements are summarized in Table 1.

For the plate containing 18 piezoceramic elements, measurements performed using the Agilent 34461A Digital Multimeter indicated an rms voltage of 30 V and an rms current of 180 µA when a pulsating force of approximately 45–47 N was applied. Under these conditions, the resulting maximum electrical power was 5.4 mW.

For the plate containing nine piezoceramic elements, an rms voltage of 22 V and an rms current of 108 µA were measured at an applied force of 42 N. The maximum power obtained in this case was 2.37 mW. For an average applied force in the range of 25–30 N, the generated electrical power was observed to vary between 1 and 1.5 mW.

The electrical response of the piezoelectric generator was characterized under different manually applied pulling forces. The measurements indicate a clear increase in both the voltage and current amplitudes as the excitation force increases.

**Figure 11:**
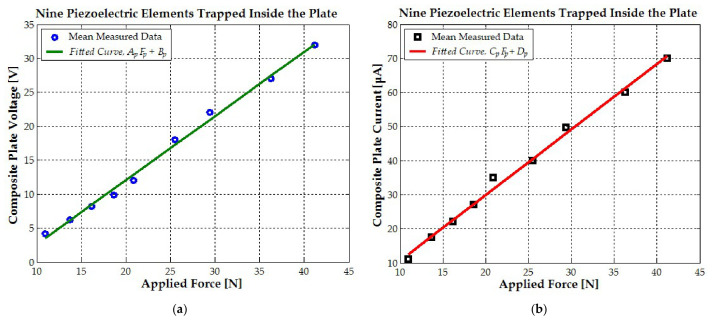
Composite plate with nine piezoelectric elements: (**a**) output voltage as a function of the pulling force; (**b**) output current as a function of the pulling force.

When a pulling force of approximately 11 N was applied on the plate containing the nine piezoelectric elements, the generated voltage pulse reached a maximum amplitude of 4.2 V, while the corresponding current pulse was about 11 µA. Increasing the force to approximately 16 N resulted in a voltage amplitude of 8.2 V and a current of 22 µA, demonstrating an almost proportional increase in electrical output with mechanical excitation (see Figure 11a,b).

For a short excitation interval of approximately 60–80 ms, applying a pulling force of about 21 N produced a voltage pulse of 12 V and a current peak of 35 µA. This indicates that stronger transient bending of the plate significantly enhances the instantaneous electrical output.

Additional measurements further confirm this trend. For instance, forces of 13.7 N and 18.6 N generated voltage amplitudes of 6.26 V and 9.85 V, with corresponding current pulses of 17.5 µA and 27 µA, respectively. At higher excitation levels, forces of approximately 29–36 N produced voltage amplitudes between 22 V and 27 V, with current pulses increasing to 49.7 µA and 60 µA. The maximum applied force of 41 N produced a voltage pulse of 32 V and a current pulse of 70 µA. The maximum power of 2.24 mW was obtained for the plate with nine piezoelectric elements.

The experimental results demonstrate a monotonic increase in both voltage and current pulses with increasing mechanical force, confirming that the electrical output of the piezoelectric harvesting structure is strongly dependent on the magnitude of the applied mechanical excitation. The response remains consistent with the expected behavior of piezoelectric transducers, where larger bending strains lead to higher generated charge and consequently higher voltage and current pulses.

**Figure 12:**
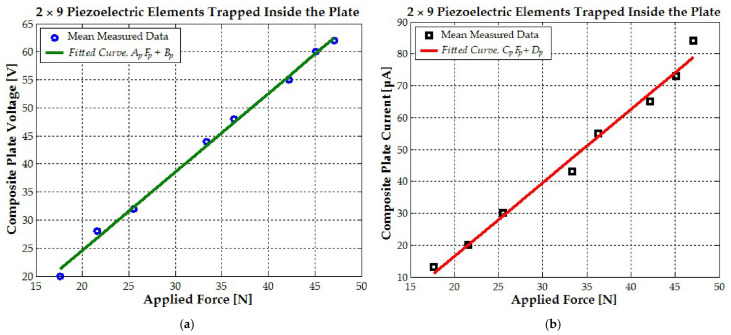
Composite plate incorporating 18 piezoelectric elements: (**a**) output voltage versus pulling force; (**b**) output current versus pulling force.

For a pulling force of approximately 17–18 N that was applied on the plate containing the 18 piezoelectric elements, the generated voltage pulse reached a maximum amplitude of 20 V, while the corresponding current pulse was around 13 µA. When the force was increased to approximately 26 N the voltage reached 33 V and the current 30 µA. At higher force levels the proportional increase becomes more pronounced. For example, forces of approximately 36 N and 42 N produced voltage peaks of 47 V and 55 V, respectively, and current peaks of 53 µA and 67 µA (see Figure 12a,b).

Voltage and current can be further estimated from the measurements by using the linear fitted model $U_{p}=A_{p}F_{p}+B_{p}\;[V]$ and $I_{p}=C_{p}F_{p}+D_{p}\;[\mu A]$ (Table 2).

For the linear fitting voltage model of the plate incorporating nine piezoelectric elements, the coefficient of determination (R^2^) was 0.9948, with a linear correlation coefficient (R) of 0.997. The corresponding relative error was approximately 5%. For the linear fitting current model, the coefficient of determination was 0.994 and the correlation coefficient was 0.997, with an average relative error of about 4%.

For the plate with 18 elements, the linear fitting voltage model exhibited a coefficient of determination of 0.9972 and a correlation coefficient of 0.9986, with a low average relative error of approximately 2%. For the current model, the coefficient of determination was 0.9874, and the correlation coefficient was 0.9937, with an average relative error of about 5%. Although these errors are comparable to the relative standard deviations obtained from the measurements, the linear fitting procedure was applied to refine the mean experimental values and ensure consistency with piezoelectric theory, which predicts a linear proportionality between generated voltage and applied force through the piezoelectric coefficient *d*.

**Figure 13:**
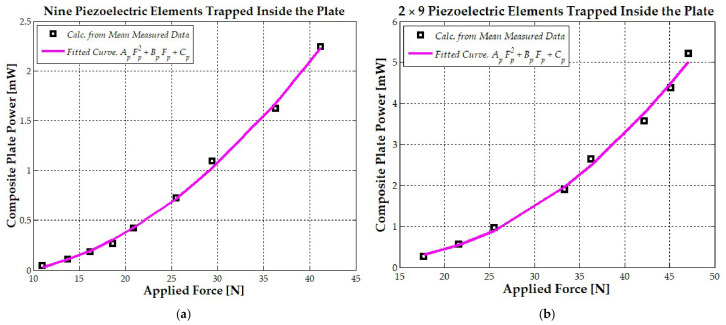
Harvested power as a function of pulling force applied to the composite plate: (**a**) configuration incorporating nine piezoelectric elements; (**b**) configuration incorporating eighteen piezoelectric elements.

The experimental measurements indicate that the generated power increases progressively with increasing pulling force. For instance, when the applied force is approximately 11 N, the generated power is close to 0.025 mW. Increasing the excitation force to around 16 N results in a power level of approximately 0.19 mW, while for a force of about 21 N, the generated power rises to roughly 0.43 mW (Figure 13a).

At higher excitation levels the increase becomes more pronounced. For example, forces of approximately 29 N, 36 N, and 41 N produce electrical power levels of approximately 0.98 mW, 1.64 mW, and 2.2 mW, respectively (Figure 13a). These results confirm a clear nonlinear growth of harvested power with the applied mechanical force. Overall, the quadratic approximation captures well the observed trend and reflects the fact that stronger mechanical excitation produces larger structural deformation and consequently higher electrical energy generation in the piezoelectric harvesting structure.

Two investigations were conducted to evaluate the dependence of the generated electrical power on the applied pulling force for both configurations, the configuration consisting of nine piezoelectric elements and the one consisting of 18 piezoelectric elements. The experimental results show that the harvested power increases nonlinearly with the applied mechanical force and can be approximated by a quadratic relationship of the form:

(4) $$P_{p}=A_{p}F_{p}^{2}+B_{p}F_{p}+C_{p}$$

This expression provides a good approximation of the measured data within the investigated force range. Coefficients $A_{p},\;B_{p},\;C_{p}$ obtained from the experimental fitting are presented in Table 3.

This quadratic relation (4) describes the nonlinear increase in the harvested power with the applied mechanical force. According to this model, the generated power rises from approximately 0.20 mW at 11 N to about 0.25 mW at 16 N, and further increases to roughly 0.5 mW at 21 N. At higher excitation levels the power grows more significantly, reaching approximately 1.3 mW at 29 N, 2.43 mW at 36 N, and about 3.48 mW at 41 N (Figure 13b: 18 piezo-element structure).

Comparing the two configurations shows that increasing the number of piezoelectric elements from nine to 18 leads to an increase in the maximum harvested power from approximately 2.2 mW to about 3.48 mW, corresponding to an improvement of 63%.

For the configuration consisting of 18 piezoelectric elements, the harvested electrical power increases with the applied mechanical force and reaches a maximum value of approximately 5.2 mW at a pulling force of 47 N. This value represents the highest electrical output obtained during the experimental investigation and reflects the combined contribution of all active piezoelectric sources under strong mechanical excitation.

In comparison, the configuration containing nine piezoelectric elements produced a lower maximum power under similar excitation conditions. At the highest investigated force level (approximately 41 N), the generated electrical power reached about 2.2 mW.

The comparison between the two configurations shows a significant improvement in the maximum harvested power when the number of piezoelectric elements is increased. Specifically, the 18-element structure generates approximately 5.2 mW, whereas the nine-element configuration produces about 2.2 mW, indicating an increase of roughly 170% (about 2.7 times higher power). This improvement can be attributed to the larger number of piezoelectric transducers contributing to the total generated charge and electrical output.

Overall, the results confirm that increasing the number of piezoelectric elements substantially enhances the maximum power that can be harvested from the mechanical excitation, although the increase is influenced by the mechanical strain distribution and structural coupling within the system.

### 3.2. Theoretical and Experimental Investigation of the Thermoelectric Device

The aluminum tube was incorporated into the thermoelectric device to contain water or other phase-change materials (PCMs), with the purpose of maintaining the thermal gradient between the cold and hot sides for as long as possible. It is expected that further insulating the ends of the device with caps around the copper sheet, together with polyurethane foam insulation, would extend both the temperature gradient $∆T_{m}$ and the thermal response time of the cold side.

The first experiment described in this study was conducted to investigate heat transfer theory and thermoelectric governing equations under simplified modeling assumptions, leading to the formulation of a general coupled system of equations describing heat transfer and thermoelectric effects.

The thermoelectric device was heated to 38–40 °C using a heating blanket placed over the copper sheet. At the same time, once the hot-side temperature reached the desired level, ice-cold water (1–3 °C) or cold water (5–10 °C) was introduced into the central aluminum tube. Afterward, the heating was stopped, allowing the device to gradually cool down until thermal equilibrium was reached.

**Figure 14:**
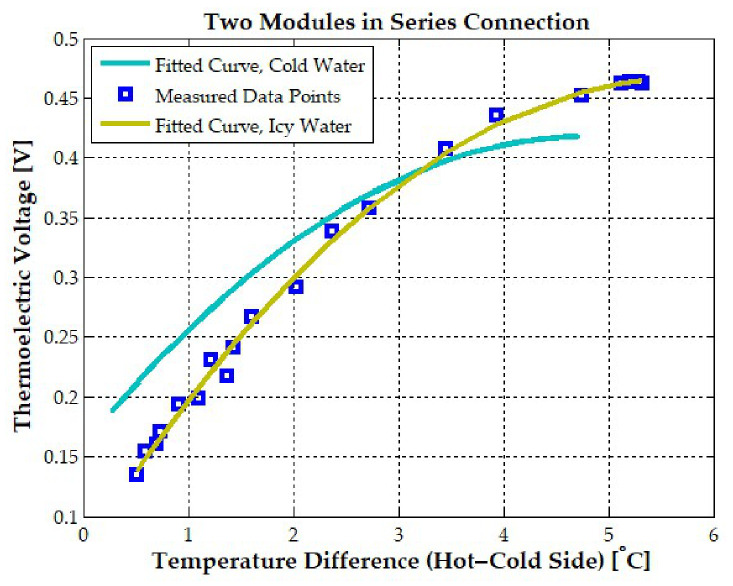
Thermoelectric voltage as a function of the temperature difference between the heated hot side and the cold side, when icy water or cold water is injected into the tube.

From the fitted voltage curves, it can be clearly observed that as the system cools, the temperature gradient $∆T_{m}$ decreases from 5.3 K to 0.5 K when icy water is injected, and from 4.6 K to 0.7 K when cold water is used. Correspondingly, the voltage decreases from 0.463 V to 0.135 V for the icy-water case and from 0.425 V to 0.21 V for the cold-water case (see Figure 14). The two curves intersect at approximately 3.2 K, where the voltage is about 0.39 V. At a temperature gradient of 2 K, the voltage is approximately 0.30 V for icy water and 0.33 V for cold water.

**Figure 15:**
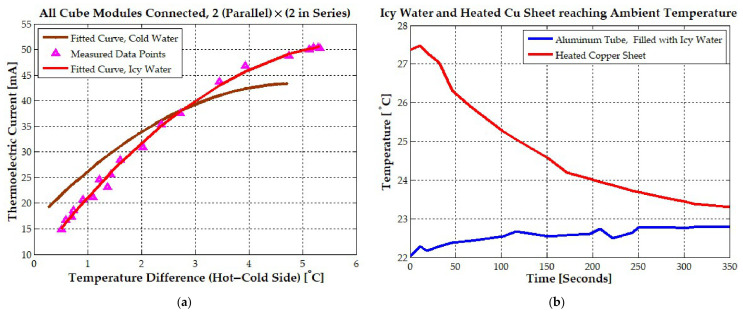
(**a**) Thermoelectric current as a function of the temperature gradient between the heated hot side and the cold side, when icy water or cold water is introduced into the tube; (**b**) temperature difference decay during system cooling for the ice-cold water case.

The current decreases correspondingly from 50 mA to 15 mA for icy water and from 44 mA to 21 mA for cold water. The two curves intersect at approximately 2.75 K, where the current is about 38 mA. At a temperature gradient of $∆T_{m}=2\;K$, the current is approximately 31 mA for icy water and 34 mA for cold water (see Figure 15a). For the icy-water case, the temperature gradient $∆T_{m}$ decreases from 5.3 K to 0.5 K, as shown in Figure 15b.

The equivalent thermal resistance of the system, $R_{sys},$ is defined as the sum of all relevant contact thermal resistances, $R_{cont}$, the thermal resistances of the copper sheet and aluminum tube, ${R_{Cusheet}\;and\;\;R}_{Altube}$, the water convection thermal resistance, $R_{wconv}$, and the equivalent parallel thermal resistance of thermoelectric modules $R_{TEP}$ and $R_{sys}=R_{Cusheet}+R_{TEP}+R_{cont}+R_{Altube}+R_{wconv}$.

From the above assumptions, the relation between junction temperature (between thermoelectric legs) and measured temperature can be defined as:

(5) $$∆T_{m}=∆T_{j}R_{sys}/R_{TEP}≅∆T_{j}\left(1+R_{wconv}/R_{TEP}\right)$$

In general, the thermal resistances of the copper sheet and the aluminum tube are extremely small, i.e., $R_{Cusheet},\;R_{Altube}<0.0005\;K/W$, and may therefore be neglected in the thermal model. The thermal resistance of the aluminum tube was estimated on the basis of its geometric dimensions, namely a length of 350 mm, lateral square sides of 18.6 mm, and a wall thickness of 2.3 mm, the initial outer square side being 19–20 mm. An aluminum pipe wall thermal conduction resistance of 0.00042–0.0005 K/W was calculated considering an area of 0.020–0.027 m^2^. The square copper sheet (outer side 62 mm, thickness 1 mm, length 250 mm) has a large lateral heat transfer area of approximately 0.061 m^2^. Combined with copper’s high thermal conductivity (~400 W/m·K), which is roughly twice that of aluminum (~205 W/m·K), the resulting conduction resistance is very low, approximately 0.000041 K/W. This is about an order of magnitude lower than the corresponding aluminum case, primarily due to the higher conductivity and slightly larger effective heat transfer area.

Because the heat flow is divided along parallel paths through all 16 thermoelectric plates $\left(N=16\right)$, corresponding to the four cubes with four active faces each, the thermal resistance of the heat sink, $R_{{HS}_{i}}=R_{HS}=7.6\;K/W$, has to be added in series to the thermal resistance of each thermoelectric module, $R_{TE,i}=R_{TE}$, as expressed by the following formula:

(6) $$R_{TEP}={\left(\sum_{i=1}^{N}\frac{1}{R_{{TE}_{i}}+R_{{HS}_{i}}}\right)}^{-1}=\frac{R_{TE}+R_{HS}}{N}$$

The convective thermal resistance on the water side can be minimized by forcing the water circulation within the aluminum tube. Increasing the flow velocity enhances convective heat transfer, thereby decreasing the associated thermal resistance. For forced water convection, the thermal resistance generally falls within the range of $R_{wconv}=0.01\ldots 0.1\;K/W$. However, when the water is static inside the aluminum square tube, heat transfer is governed by much weaker natural convection mechanisms, and the thermal resistance increases to approximately $R_{wconv}=0.2\ldots 0.3\;K/W$. The equivalent parallel thermal resistance of thermoelectric modules is $R_{TEP}≅0.45\ldots 0.55\;K/W$, because the internal thermal resistance of a single thermoelectric module is $R_{TE}=1.0-1.2\;K/W$.

(7) $$∆T_{j}=\frac{∆T_{m}}{1+C∆T_{m}},\;C≅\frac{R_{wconv}}{∆T_{m}R_{TEP}}$$

For both cases (static or circulating water), $C\in \left[0.01\ldots 0.6\right]$. In the present work, the water is static inside the pipe, so $C\in \left[0.2\ldots 0.6\right]$.

Parameter C can be interpreted as an external thermal feedback coefficient, which incorporates the cumulative effect of the glue-layer thermal contact, heat conduction through the ceramic layers, thermal spreading within the copper sheet and aluminum heat sink, and, most importantly, the convective heat transfer associated with the water flow. The electrical power generated within the thermoelectric module is governed by the Seebeck coefficient, the internal resistance $R_{TE}$, and the temperature difference at the BiTe junctions, $\Delta T_{j}$.

(8) $$P=A_{0}\frac{∆T_{j}^{2}}{1+B_{0}∆T_{j}},\;R_{TE}=R_{0}\left(1+B_{0}∆T_{j}\right)$$

The temperature coefficient of resistance $B_{0}\approx 0.004-0.006\;1/{}^{\circ}C$ is much smaller when compared to the external thermal feedback coefficient, $C\gg \;B_{0}$.

Since the junction temperature cannot be measured directly, relation (7) is used to determine the junction temperature difference function of temperature gradient between the aluminum cold side and the copper-sheet hot side, temperatures were measured on both sides by attaching K-type thermocouples. If we replace the junction temperature difference, $∆T_{j}$ (between cold and hot legs), with the measured temperature difference, $∆T_{m}$, between the aluminum tube and the copper outer sheet, the power relation becomes:

(9) $$P=A_{0}\frac{∆T_{m}^{2}}{\left(1+C∆T_{m}\right)\left[1+\left(B_{0}+C\right)∆T_{m}\right]}≅A\frac{∆T_{m}^{2}}{\left(1+B∆T_{m}+D∆T_{m}^{2}\right)}$$

In order to satisfy both denominators, the equality between coefficients must be ${A=A}_{0},\;B=B_{0}+2C,\;D=C\left(B_{0}+C\right)$. Because $C\gg \;B_{0}$, B≅2C.

The global thermal feedback coefficient B is a lumped, system-level parameter that quantifies nonlinear coupling between electrical transport, internal heat generation, and external thermal boundary conditions. The thermal feedback coefficient is not considered a pure material constant. The thermal feedback coefficient of the system B is influenced mainly by electrical resistance growth, internal Joule heating, Seebeck nonlinearity, external thermal resistances, water convection nonlinearity and contact resistances. The thermal feedback coefficient B accounts for the influence of external thermal and electrical constraints that progressively drive the system toward saturation as the temperature difference increases. In contrast, the coefficient A characterizes the intrinsic power scaling, representing the quadratic dependence of output power on temperature difference as governed primarily by the Seebeck effect and the resulting thermoelectric voltage growth.

A quadratic polynomial and a first-order rational function have identical behavior up to second order if coefficients are matched. The rational form is simply a compact re-parameterization of the same local Taylor expansion. That means $P=A\frac{∆T_{m}^{2}}{\left(1+B∆T_{m}+D∆T_{m}^{2}\right)}≅\frac{A_{1}∆T_{m}}{1+B_{1}∆T_{m}}$ because $\left|D∆T_{m}^{2}\right|\ll B∆T_{m}$, and the first order rational function is Padé 1/1 approximant only for small temperature differences,$\;∆T_{m}<10\;K$.

**Figure 16:**
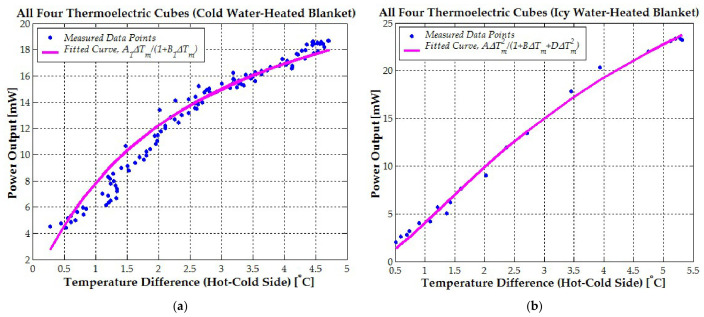
Harvested power of the thermoelectric device as a function of the temperature difference: (**a**) with a heating blanket and cold water injected into the tube; (**b**) with a heating blanket and icy water injected into the tube.

From Figure 16a the first order rational function coefficients are: $A_{1}=4\times 2.75\times 10^{-3}\;\frac{℃}{W},\;B_{1}=0.4\;\frac{1}{℃}$. In addition, from the second graph, Figure 16b, $A=7.5865\times 10^{-3}\;℃/W$, B=0.75 1/℃ and $D=0.1433\;1/{\left(℃\right)}^{2}\;$, coefficients are corresponding to the second order rational function.

By substituting the above coefficient values into relation (9), taking into account the specified equalities between the coefficients, and by slightly adjusting the value of coefficient *D* in order to obtain real solutions for *C*, the following equation describing the device behavior is obtained.

(10) $$C^{2}-0.75C+0.140=0$$

This nonlinear rise of internal resistance and the high C=0.35…0.40 1/℃ external thermal resistance ratio of the entire device directly explains the saturation behavior in the power output curve, as in Figure 16b. Since the maximum power is given by relation (9), the numerator increases quadratically with measured temperature difference Δ*T_m_*, whereas the denominator transitions from quasi-linear to super-linear growth beyond Δ*T_m_* ≈ 3 °C. Consequently, at small temperature differences, power increases rapidly due to the dominance of the Seebeck voltage term. At higher Δ*T*, the accelerated growth of internal resistance offsets the quadratic voltage gain, leading to diminishing incremental power gains and the onset of saturation.

The first-order rational function depicted in Figure 16a demonstrates a gradual decrease in power output, from 17.95 mW at a temperature difference of 4.7 °C to 16 mW at 3.5 °C, further declining to 12.2 mW at 2 °C and 7.8 mW at 1 °C.

Similarly, the cooling experiment illustrated in Figure 16b shows a reduction in generated power from 23.7 mW, recorded at a hot-side temperature of 27.5 °C and a temperature difference of 5.3 °C, to 4 mW at a temperature difference of 1 °C.

At the midpoint of the second-order rational function, corresponding to a copper-sheet temperature of 25.5 °C and a temperature difference of 3 °C, the measured power output was 15 mW. The system required approximately 90 s of gradual cooling to reach this intermediate power level.

As a function of time, the thermoelectric device is initially heated to 38–40 °C using a heating blanket covering the copper sheet; once the hot side reaches the target temperature, cold or icy water is injected into the central aluminum tube with a graduated syringe, the heat supply is removed, and the system evolves naturally toward thermal equilibrium. Depending on the initial conditions, this evolution toward thermal equilibrium may correspond either to a cooling process or to the reverse heating process. In both cases, the temperature of the hot side is reaching the same value of 28–29 °C, due to copper sheet heat spreading after approximately 7 min.

**Figure 17:**
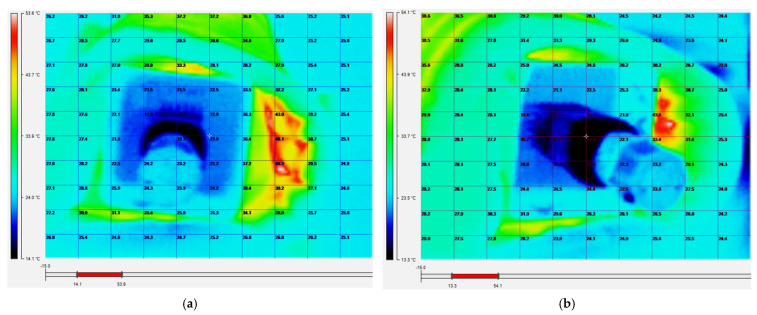
Infrared thermography of a thermoelectric device under heating blanket and with icy water poured inside the central aluminum tube: (**a**) front and (**b**) lateral views at successive time intervals.

The Ti20 thermal imaging camera and the InsideIR thermal analysis software were utilized to perform an in-depth thermographic characterization of the thermoelectric heat-transfer assembly. The thermographic analysis from Figure 17a,b revealed a strong radial temperature gradient within the thermoelectric heat-sink assembly, with temperatures ranging from approximately 15 °C at the central aluminum cooling tube to 27–31 °C near the externally heated copper surface. The heating blanket exhibited surface temperatures ranging between 38 and 44 °C, while localized regions positioned farther from the copper shell with round edges exceeded 46 °C. The outer copper sheet exhibited relatively uniform heat spreading due to its high thermal conductivity, while localized hotspots indicated regions of nonuniform thermal contact between the heating blanket and copper sheet surface.

A significant temperature drop was observed across the 4 mm thermoelectric layer, confirming effective heat transfer from the heated copper plate toward the cold-side heat sink. The thermoelectric modules established a thermal bridge between the hot external surface and the internally cooled structure, producing an estimated temperature differential of 10–15 °C across the device thickness. No abnormal heat leakage was observed through the epoxy putty or polyurethane thermal insulation separating the aluminum cubes from the thermoelectric elements, despite the possibility of parasitic heat transfer arising from their close physical contact.

The 16.5 mm heat-sink region displayed intermediate temperatures between 23 and 16 °C, demonstrating efficient thermal conduction toward the central cooling zone. While the base of the heat-sink (4 mm) showed temperatures between 21 and 23 °C, the fin structure showed progressive cooling from the fin bases to the fin tips, with temperatures decreasing from approximately 21 °C to 16–18 °C near the aluminum tube interface. This behavior indicates substantial axial heat transfer along the fins toward the cold sink.

The aluminum tube filled with icy water represented the coldest region of the system, with measured surface temperatures between 14 and 18 °C. The concentrated dark-blue thermal region surrounding the tube confirmed strong heat extraction capability and effective removal of thermal energy from the thermoelectric assembly. Overall, the thermographic field demonstrated successful inward heat transport from the heated copper shell toward the static water-cooled aluminum core, while also revealing localized asymmetries associated with thermal contact resistance and nonuniform thermal distribution. The infrared (IR) thermographic imaging is consistent with previously measured experimental data obtained for the thermoelectric device copper shell covered with the heating blanket and icy water cooling configuration. The infrared thermal analysis additionally provided the missing thermal data associated with the heating blanket, showing surface temperatures ranging between 38 and 44 °C.

As shown in the following results, heating the copper sheet while introducing cold water into the aluminum tube leads to a shorter transient time to thermal equilibrium compared with the case in which the entire device cools naturally to equilibrium with the ambient environment.

Once the hot side of the thermoelectric device stabilizes at ambient temperature (19–21 °C), a K-type thermocouple is fixed to the copper sheet and ice-cold water, at 2–5 °C, is introduced into the central aluminum tube. Following this step, the copper sheet is allowed to continue cooling until thermal equilibrium is established. Simultaneously, the temperature of the aluminum tube rises gradually from 16.5 °C to the equilibrium temperature of 19.8 °C. The experimentally measured time for this slow warming process is 11 min 33 s.

During the initial 5 min of the transient regime, the temperature difference across the device decreases from 3.3 °C to 0.7 °C. Correspondingly, the output power drops from a maximum measured value of 2.64 mW to 0.4 mW, with the cold-side temperature reaching 18.7 °C. At this point, the output voltage is only about 62 mV, and the electronic part cease to operate.

**Figure 18:**
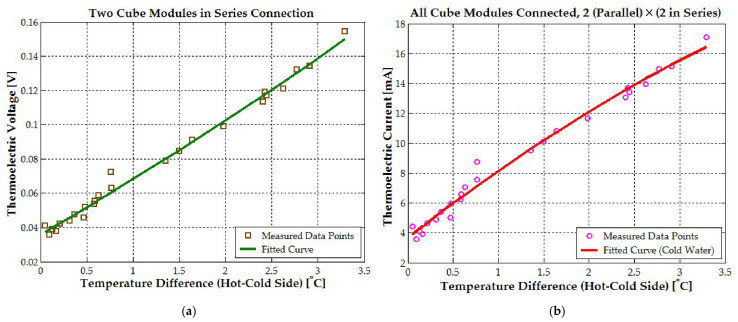
Thermoelectric response to temperature difference: (**a**) thermoelectric voltage as a function of the temperature difference, with ice-cold water introduced into the tube; (**b**) thermoelectric current as a function of the temperature difference between the hot side and the cold side.

At a temperature difference $∆T_{m}$ of 3.3 °C, a single cube-type thermoelectric module generated a voltage of 77 mV. Each module, as described earlier, consisted of four 40 × 40 mm thermoelectric plates connected in series. When two cube-type modules were connected in series, the output voltage increased to 154 mV, as presented in Figure 18a. At the same temperature gradient, the measured current was 17 mA for two pairs of cube-type series modules connected in parallel (four modules).

At a reduced temperature gradient $∆T_{m}$ of 2 °C, the voltage generated by each module decreased to 50 mV, while the current decreased to approximately 6 mA. As a result, for the complete arrangement of four cube-type modules connected as two series pairs in parallel, the total output voltage reached 102 mV, and the generated current was 12 mA, as shown in Figure 18b.

At a temperature gradient $∆T_{m}$ of 1 °C, the measured voltage and current were 68 mV and 8.1 mA, respectively. In this case, the overall electrical power generated by the four interconnected cube-type modules was 0.55 mW, with the cold-side temperature equal to 18.4 °C.

**Figure 19:**
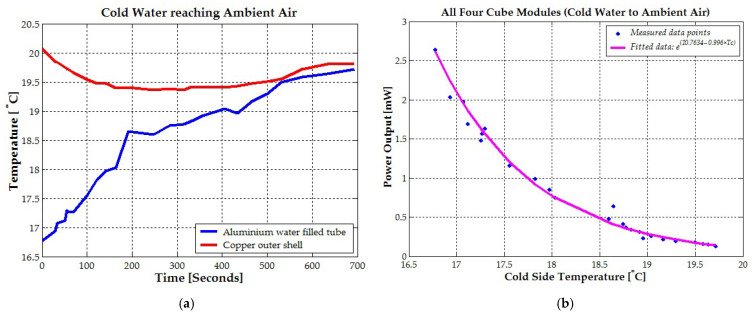
(**a**) Temporal variation of the temperature difference until the ice-cold water approaches thermal equilibrium with the ambient environment; (**b**) power generated by all cube-type modules for the same operating conditions.

Within approximately 100 s, the surface temperature of the aluminum tube (the cold side) increases by 1.3 °C, reducing the temperature difference to 2 °C, as shown in Figure 19a. Under these conditions, the generated power is 1.23 mW, at a cold-side temperature of 17.5 °C. When the tube is filled with ice-cold water, the generated power remains within the range of 2.1–2.6 mW until the tube temperature reaches 17 °C. Although this time interval is relatively short, only 33 s, the maximum useful power is obtained in this case, as illustrated in Figure 19b. Therefore, the thermoelectric device can generate an average power of approximately 2.3 mW for about 30 s solely by introducing ice-cold water into the aluminum tube, while the temperature of the hot side, represented by the copper sheet, is initially approximately equal to the ambient temperature, namely 20 °C.

**Figure 20:**
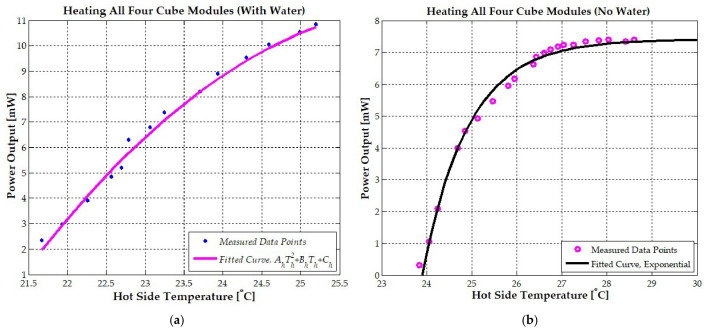
Comparison of generated power under wet and dry thermal conditions: (**a**) variation of the generated power with hot-side temperature during heating, with cold spring water injected into the tube; (**b**) generated power during heating under dry-tube conditions, with no water inside the tube.

The thermoelectric device is heated to 24–26 °C either by direct contact with the human body or by means of a heating blanket covering the entire copper sheet. A K-type thermocouple is attached to the copper sheet in order to monitor the hot-side temperature. At the beginning of the heating process, spring water at 10–12 °C is introduced into the central aluminum tube, as presented in Figure 20a. The ambient temperature during the experiment is 22 °C.

In the second experimental scenario, the aluminum tube remains empty, with no water introduced. In this case, the hot side of the thermoelectric device is initially at approximately 23 °C, close to ambient temperature, and then increases gradually under body heating to 28–29 °C over a period of several minutes, as shown in Figure 20b. The thermal behavior observed in both experiments can be represented satisfactorily by quadratic or exponential fitting functions.

For the first heating experiment, the relationship between generated power and hot-side temperature is described by a quadratic fit of the form given by the experimental data, with coefficients $A_{h}=-0.000383\;W{/}^{\circ }C^{2}$, $B_{h}=0.020435\;W{/}^{\circ }C$, and $D_{h}=-0.261\;W$. According to the measurements, the generated power reaches 10.87 mW at a hot-side temperature of 25 °C, while at 22.2 °C, the measured power is approximately 4 mW.

The quadratic fit reproduces the experimental power data with good accuracy; nevertheless, it does not capture a saturation behavior, but rather represents only a maximum in the power output and a possible decline beyond that point.

In contrast, the heating experiment presented in Figure 20b exhibits an exponential increase in generated power, as described by Equation (11). The output power increases from 1 mW at a hot-side temperature of 24 °C and approaches saturation at 29 °C, where a maximum value of 7.3 mW is obtained. At the midpoint of the exponential trend, corresponding to a copper-sheet temperature of 25 °C and an aluminum-tube temperature of 22.94 °C, the measured power output is 5 mW.

(11) $$P_{hnw}=0.0074\left(1-e^{-0.98\left(T_{h}-23.9\right)}\right)$$

**Figure 21:**
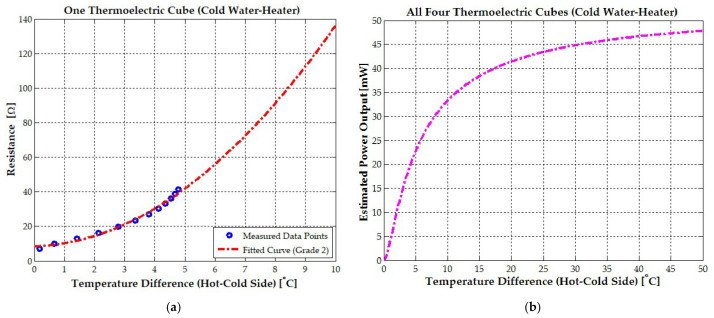
Electrical performance of a cube-type thermoelectric module under varying thermal conditions: (**a**) internal electrical resistance of a single cube-type thermoelectric module as a function of temperature difference; (**b**) estimated power output over an extended temperature-difference range under conditions of cold-water cooling and different heating sources.

The first graph in Figure 21a shows that the internal electrical resistance of a single cube-type thermoelectric module increases nonlinearly with the temperature difference, ΔT. When ΔT exceeds 3 °C, the variation can be approximated by a second-order dependence, while for very small temperature differences, in the range 0 °C<ΔT<3 °C, the behavior is quasi-linear. For temperature differences close to zero, the internal resistance of the complete thermoelectric cube, composed of four thermoelectric plates connected in series, ranges from 6 to 7 Ω. This arrangement is supported by the presence of the copper sheet, which provides a uniform heat distribution over all thermoelectric modules. In this case, the resistance of a single thermoelectric plate can be considered 1.6 Ω. At a temperature difference of 0.18 °C, the measured internal resistance is 7.13 Ω. The internal resistance ($R_{TE}$) estimated from Equation (8) is approximately four times lower than the experimentally measured internal resistance of the series-connected TEC1-12706 modules of the thermoelectric cube. The quasi-linear rise in resistance, from 7.13 Ω to 19.88 Ω, is limited to the interval 0–2.78 °C. Beyond this range, the increase becomes more pronounced: between 3 and 5 °C, the internal resistance grows quadratically from 23 Ω to 42 Ω. For temperature differences above 10 °C, the estimated internal resistance reaches about 140 Ω, which significantly restricts the generated current, although the voltage output is higher.

The observed saturation in power output with increasing temperature difference is directly attributable to the nonlinear increase in internal electrical resistance (Figure 21a,b). The same coefficients used in Figure 16b were also applied to Figure 21b.

The results shown in these plots are among the most relevant experimental findings, since they represent the maximum achievable temperature gradient in the proposed system, with the hot side operating near body temperature and the cold side maintained at the temperature of icy water.

The first-order rational function shown in Figure 16a reaches saturation at approximately 25 mW, occurring at a relatively low temperature difference of 20 °C.

In contrast, the second-order rational function presented in Figure 16b and Figure 21b provides a more accurate fit to the experimental data, with the saturation curve stabilizing at around 50 mW for temperature differences exceeding 27 °C. Notably, the power output increases more rapidly with temperature difference up to approximately 15 °C, reaching about 38 mW. Beyond this point, the rate of power increase diminishes significantly, indicating a reduced incremental gain despite further increases in temperature difference.

As internal resistance rises with temperature difference between hot and cold side, the denominator in the power relation (9) increases sufficiently to offset the quadratic growth in Seebeck voltage, resulting in diminishing power gains and eventual saturation.

### 3.3. Electronic Energy Management and Storage Circuits

**Figure 22:**
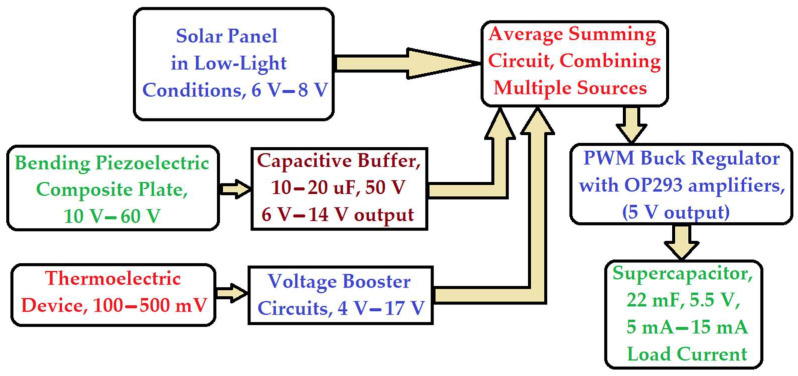
Block diagram of the electronic energy management circuits.

The electronic management stage was designed to combine intermittent and low-current outputs from the piezoelectric, thermoelectric, and photovoltaic sources into a regulated storage voltage suitable for supercapacitor charging (Figure 22). The thermoelectric voltages were first increased by two Meissner oscillator circuits, each connected to a pair of thermoelectric cube modules. The boosted and rectified thermoelectric voltages were then combined with the rectified and filtered piezoelectric output and the photovoltaic contribution through a high-impedance averaging and decoupling network. This stage reduces the direct interaction between sources with different internal resistances and temporal characteristics. The resulting voltage is applied to an ultra-low-power PWM buck regulator based on OP293 operational amplifiers and a low-resistance PMOS switching transistor. The regulated 5 V output charges a 22 mF supercapacitor, while a 5.6 V low-leakage Zener diode limits overvoltage across the 5.5 V-rated storage element. This architecture allows intermittent harvested energy to be accumulated safely and converted into a more stable output suitable for low-power electronic loads [29,30].

**Figure 23:**
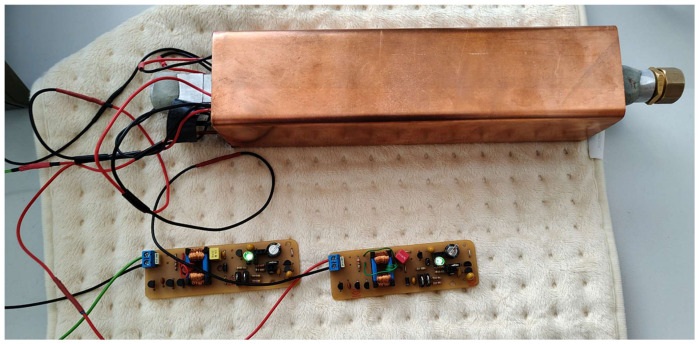
Thermoelectric harvesting device comprising four thermoelectric cubes, arranged as two series-connected pairs, each powering a Meissner oscillator circuit and a signaling green LED.

Two similar Meissner oscillator circuits were built, each connected to two TEGs placed in series on one side of the thermoelectric device. This approach was necessary because the heat delivered by the palms cannot uniformly cover the entire 250 mm-long copper sheet (see Figure 23).

By dividing the device into two active thermoelectric sections, the available body heat could be exploited more effectively, and each TEG pair could operate under more uniform thermal conditions. The corresponding Meissner oscillator circuits were used to process independently the very low voltages generated by each TEG pair and to boost them to a level suitable for the following stage [30]. The circuit is based on two parallel 2SK117 JFETs, used to share the input current and reduce the equivalent dynamic drain resistance, thereby limiting conduction losses, and on a BS170 MOSFET, which takes over the current once the oscillation amplitude becomes sufficiently high [30]. Using a transformer ratio of 1:48, the circuit can raise an input voltage of 0.1 V to as much as 4 V and an input voltage of 0.48 V up to 17 V. Output voltage was measured on transformer secondary between the rectifying PMEG4005EH or SB140 Schottky diode positive side and ground. The 1–2 μF capacitor placed at the transformer secondary ensures output filtering and provides a smoother DC voltage for the power-management stage.

At peak thermoelectric power generation, corresponding to a hot-side temperature of 30 °C and a cold-side temperature of 22 °C, the circuit boosts an input voltage of approximately 0.48 V to as high as 17 V. As the device cools, the input voltage gradually decreases to 0.45 V, resulting in an output voltage of 15.4 V. With further cooling, the input–output voltage pairs reduce to 0.41 V and 14.1 V, and subsequently to 0.30 V and 10.5 V.

**Figure 24:**
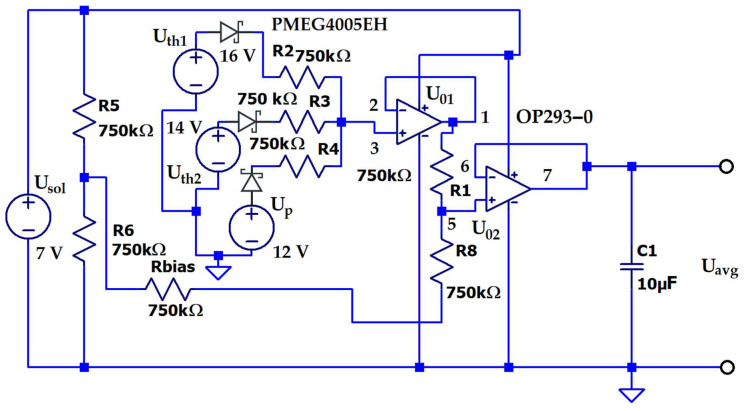
Schematic of the average voltage summing, source isolation and decoupling circuit for combining multiple low-power energy sources—namely, solar, thermoelectric, and piezoelectric generators—exhibiting complementary characteristics.

**Figure 25:**
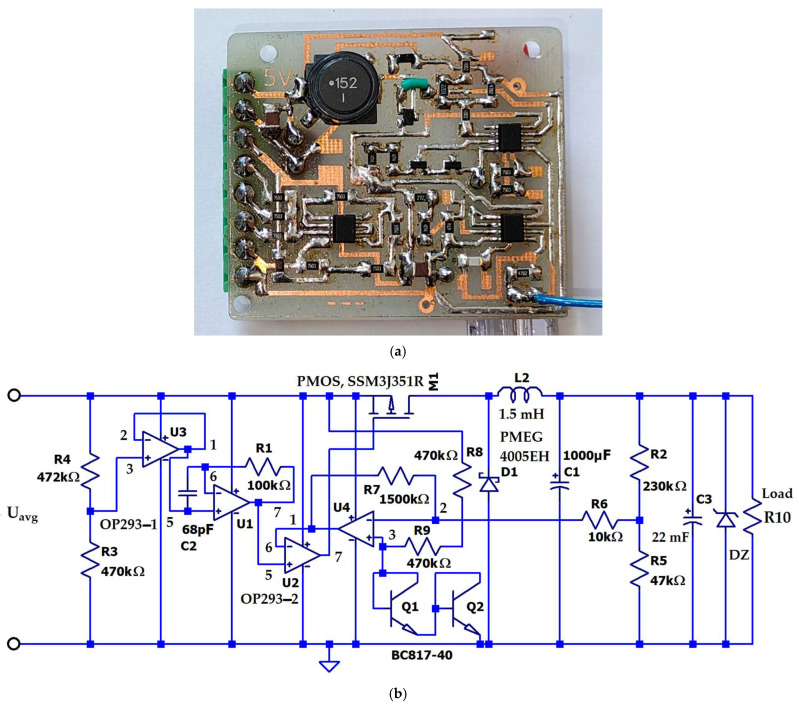
Printed circuit board (PCB) implementing an average voltage summing circuit for multiple low-power energy sources with complementary characteristics, together with an ultra-low-power 5 V voltage regulator: (**a**) average voltage summing and voltage regulation circuits with surface-mounted (SMD) components; (**b**) fully automated ultra-low-power 5 V voltage regulator scheme realized using two OP293 operational amplifier devices.

The thermoelectric source voltages, $U_{th1}$ and $U_{th2}$, are derived from the secondary windings of the previously described Meissner oscillator circuits. These voltages are summed together with the piezoelectric source voltage, $U_{p}$. The resulting signal, filtered by a 10 µF ceramic multilayer capacitor, represents the averaged sum of half the solar panel voltage and the individual sources contributions (Figure 24). The electrical symbols and numerical labels in the Figure 24 and Figure 25 were automatically generated by LTspice software (version 24.0.9).

Both amplifiers (U01 and U02) within the Analog Devices OP293–0 are configured as voltage followers, with their inverting inputs (pins 2 and 6) connected directly to their respective outputs (pins 1 and 7). In this configuration, U02 operates as a buffer stage to ensure proper biasing and signal stability. Its non-inverting input is connected to the summing node at the output of the first amplifier (U01). Resistors R1 and R8 are used to further adjust the output voltage (if needed). Here, no further adjustments are needed, so R1 and R8 are equal. All average summing circuit resistances have the same value of 750 kΩ. From the above assumptions, the average voltage summing is defined by the following relation:

(12) $$U_{avg}=\frac{U_{sol}/2+U_{th1}+U_{th2}+U_{p}}{4}$$

Resistors R5 and R6 establish a reference voltage (virtual ground) equal to one-half of the average solar panel voltage, $U_{avg}$. The output of the first amplifier (U01) within the Analog Devices OP293–0 is biased with respect to this reference level.

The “Star Solar” (Guangzhou) polycrystalline panel (125 × 195 mm) provides a nominal voltage of approximately 9 V at maximum power. Under full illumination conditions, it can deliver a current on the order of 300–350 mA. The corresponding open-circuit voltage is expected to exceed the nominal value.

The voltage regulator circuit (Figure 25a,b) incorporates a fully automated feedback control mechanism. It consists of a voltage follower (U3), which establishes a stable virtual ground, a quasi-triangle-wave generator (amplifier U1) that oscillates around this virtual reference (${U_{ref}=U}_{avg}/2$), a PWM comparator that compares the triangular waveform with the DC control voltage corresponding to the +5 V output, and an error amplifier (U4) responsible for regulating the control signal. The comparator generates a rectangular PWM signal with an appropriate duty cycle to drive the PMOS transistor.

The reference voltage is established by means of resistors R3 and R4, forming a voltage divider that defines the virtual ground. This configuration is necessary because the operational amplifier is powered in a single-supply mode (3 V to 20 V) with respect to virtual ground, rather than in a dual-supply (differential) configuration.

Resistors R2 and R5 form the feedback voltage divider for the error amplifier. The resistor values are selected based on a feedback ratio corresponding to an output voltage of 5 V and a reference voltage in the range of 0.78–0.85 V. The upper bound of 0.85 V, corresponding to higher than 10 V input voltages, is provided by a stable reference implemented using two BC817-40 NPN transistor devices configured with their bases and collectors connected, thereby operating as diode-connected transistors. This configuration establishes a relatively constant voltage drop between the collector and emitter, which serves as the reference for the regulation loop.

The error voltage ($U_{err}=U_{out}-U_{feedback}$) is defined as the difference between the reference (target) output voltage and the feedback voltage obtained from the resistive divider. This error signal is applied to the inverting input of the error amplifier (U4) of OP293−2 integrate and is subsequently amplified according to the closed-loop gain of the inverting configuration. The gain is determined by the ratio of resistor R7 (1.5 MΩ), connected between the amplifier output and the inverting input, to the equivalent input resistance formed by resistors R2 and R5 (230 kΩ and 47 kΩ, respectively). Accordingly, the control voltage and the voltage gain of the error amplifier is given by:

(13) $$U_{control}=A_{u}\cdot \left(U_{out}-U_{feedback}\right),\;A_{u}=\frac{R7}{R2+R5}$$

The negative sign indicates the phase inversion inherent to the inverting configuration. This amplified error signal defines the control voltage used by the PWM comparator, thereby regulating the duty cycle and stabilizing the output voltage.

The quasi-triangular wave generator operates as a relaxation circuit in which the timing capacitance is referenced to a constant voltage rather than ground. The resulting waveform exhibits a predominantly triangular shape with asymmetrical characteristics: a rounded upper edge caused by an exponential charging process, and a nearly linear descending edge corresponding to a quasi-constant current during the discharging phase. The slope of the output signal $U_{triangle}$ at the output of amplifier U1 is directly proportional to the current flowing through capacitor $C_{2}$, as expressed by:

(14) $$i_{C}=C_{2}\frac{d}{dt}\left(U_{triangle}-U_{ref}\right)$$

The proposed circuit achieves quasi-triangular wave generation using a single operational amplifier U1, eliminating the need for a separate Schmitt trigger, an additional integrator stage and a non-overlapping buffer [31,32]. This approach results in a more compact architecture with reduced passive and active components count, thereby simplifying implementation while maintaining the required oscillatory behavior. The voltage regulator architecture incorporates four operational amplifiers. Specifically, the odd-numbered amplifiers U1 and U3, housed within the OP293–1 package, are configured to establish a stable voltage reference and synthesize the output triangle wave. Conversely, the even-numbered units, housed by the OP293–2 package, function as a comparator (U2) and an error amplifier (U4), respectively. Within the comparator stage, the triangle wave is modulated by the DC feedback signal to generate a pulse-width modulated (PWM) waveform with the requisite duty cycle [33]. This duty cycle is dynamically adjusted based on the input-to-output voltage ratio; for instance, given an input of 15 V and a target output of 5 V, the switching transistor operates with a 25% conduction interval and a 75% cutoff period. A similar proportional adjustment, of 50%, occurs for a 10 V to 5 V conversion.

The supply current per amplifier is limited to a maximum of 30 µA. Consequently, for three Analog Devices OP293 units—comprising a total of six amplifiers—the maximum current consumption is 180 µA. This current budget accounts for both the average summing voltage circuit and the 5 V buck regulator. All resistive elements were carefully selected to minimize current flow, with operating currents constrained to the range of a few microamperes, typically between several µA and 12 µA, and not exceeding 20 µA. As a result, the combined current consumption of the averaging (summing) stage and the voltage regulation (control) circuitry remains below 300 µA. For comparison, conventional PWM drivers or integrated buck regulators such as LM25017 [29] typically exhibit supply currents on the order of 1–10 mA, highlighting the efficiency of the proposed design. The control circuitry (command block) consumes approximately 1.9 mW of power.

For applications requiring low supply and load currents, typically in the range of 1 to 200 µA, while maintaining operation at high switching frequencies, conventional discrete buck converter implementations become inefficient or impractical. In such regimes, parasitic effects, switching losses, and control overhead can dominate overall performance. Therefore, specialized buck converters must be monolithically integrated on-chip and carefully optimized for ultra-low-power operation [34,35,36]. These integrated designs often employ techniques such as pulse-frequency modulation (PFM) or hysteretic control instead of traditional pulse-width modulation (PWM), as they reduce switching activity and associated losses under light-load conditions. Additionally, on-chip integration minimizes parasitic inductances and capacitances, which is critical at high frequencies, where such non-idealities significantly impact efficiency and stability.

Furthermore, the use of high switching frequencies enables the reduction of passive component sizes, particularly inductors and capacitors, facilitating full or partial integration within the silicon die or package [37].

In the power stage, a current of 9–10 mA flows through the coil. Under these conditions, the power dissipated across the 1.9–2 Ω internal resistor is approximately 240 µW, while the conduction losses in the PMOS transistor are approximately 12 µW (0.1–0.12 Ω on resistance), corresponding to a load power transfer of about 46 mW. Based on the experimental data, it can be concluded that a total power loss of approximately 3.5–5 mW occurred within the energy management stage. Given an input power of 50 mW, this corresponds to an efficiency of at least 90% for the core of energy management circuit.

This system implements a high-efficiency buck converter designed for energy harvesting from solar (7 V), thermoelectric (14 V and 16 V) and piezoelectric (12 V) sources regulated via an OP293 ultra-low-power operational amplifier. The architecture utilizes a voltage-follower and an average summing input stage for source decoupling, feeding a PWM comparator that modulates an SSM3J351R P- MOSFET drain to source channel with R_ds_on_ = 0.1 Ω. Although the average input current is only 2–3 mA, the converter maintains a stable 5 V output, even under an inductor ripple current of over 10 mA.

The discrepancy between the low source current and high ripple current is attributed to the Discontinuous Conduction Mode (DCM) of operation [36]. The oversized 1.5 mH inductor (rated for 500 mA) acts as a high energy reservoir, while the 1000 µF output capacitor effectively filters the audible frequency signals (situated between 3 and 10 kHz). This configuration ensures that the average power delivered by the high-impedance sources is sufficient to maintain regulation, with the inductor ripple being largely reactive and contained within the output filtering stage.

Efficiency is maximized by leveraging the OP293’s microampere current, which prevents the control circuitry from exhausting the limited energy delivered by the harvesting sources. The use of a low-threshold MOSFET (Vth = 0.8 V) ensures reliable switching even at low input potentials. To protect the connected Panasonic Gold Capacitor (GC) type supercapacitor rated at 5.5 V (GC5.5V.022F), having an internal equivalent series resistance lower than 75 Ω (ESR), a low-leakage Zener diode of 5.6 V (MMSZ4690 from Vishay) is connected in parallel to prevent overvoltage conditions during transient load changes, ensuring long-term reliability of the energy storage components. The PMEG4005EH Schottky barrier rectifier diode, manufactured by Nexperia, is designed for efficient rectification in low-power applications. It supports a maximum repetitive peak reverse voltage of 40 V. A key characteristic of this diode is its exceptionally low forward voltage drop, typically ranging from 0.1 V to 0.15 V at forward currents between 0.1 mA and 1 mA. This low forward voltage contributes to reduced conduction losses and improved efficiency, particularly in energy-sensitive or low-current circuits. Because the typical junction capacitance of the PMEG4005EH diode is on the order of tens of picofarads, and due to the absence of reverse recovery effects, the device is capable of high-speed operation, with practical maximum operating frequencies on the order of MHz.

The BAS40-04 Schottky diode array, comprising two Schottky diodes connected in series, exhibits a low forward voltage drop of approximately 0.15–0.20 V per diode at the operating current range of 7–12 µA generated by each piezoelectric element under applied forces of 41–47 N. Since each full-bridge rectifier consists of four diodes, the power dissipated in a single rectifier is approximately 6 µW. Consequently, for the composite plate incorporating nine piezoelectric elements, nine full-bridge rectifiers are required, resulting in a total rectification loss of approximately 54 µW. Similarly, for the composite plate incorporating 18 piezoelectric elements, 18 full-bridge rectifiers are employed, leading to a total rectification loss of approximately 108 µW.

At the highest investigated force level of approximately 41 N, the composite plate containing nine piezoelectric elements generated a maximum electrical power of about 2.2 mW. Accounting for the rectification losses, the corresponding power conversion efficiency was approximately 97–98%. Likewise, for the configuration containing 18 piezoelectric elements, the generated electrical power reached approximately 5.2 mW at the highest investigated force level of about 47 N. With rectification losses of 108 µW, the overall efficiency remained close to 98%.

The thermoelectric source delivers a maximum input power of 25 mW to a Meissner oscillator-based resonant conversion stage, which also functions as a voltage and signal (booster) conditioning circuit. Within this stage, dissipative losses associated with active switching devices (BS170 MOSFETs/2SK117 JFET biasing network) and transformer core and copper losses are estimated in the range of 6–9 mW. Consequently, the net power delivered to the load is approximately 16–19 mW, corresponding to an overall electrical conversion efficiency of 64–76% for the Meissner oscillator circuit under the specified operating conditions. If the previously calculated efficiencies are multiplied, 0.98 × 0.90 × 0.69 ≈ 61%, which is the overall (rough system-level estimate of) typical electronic-path efficiency.

The regulated 5 V output was connected to one of the two 22 mF supercapacitors. Under these conditions, both the supercapacitor and the 1000 µF electrolytic capacitor at the output reached full charge (at 5 V) in approximately 100 s. The 1000 µF electrolytic capacitor alone charged within 15–20 s, depending on the current supplied by the energy sources. The experimental results indicated that the supercapacitor reached approximately 2 V within 25–30 s and approximately 4 V after 1 min of charging.

The analysis considers an optimal operating scenario in which the combined contribution of thermoelectric, piezoelectric, and photovoltaic sources yields a total power output of approximately 43–45 mW. Prior experimental investigations on the thermoelectric subsystem demonstrated that four thermoelectric cube modules can generate between 20 and 25 mW. The photovoltaic source contributes at least 14 mW under natural daylight conditions, including cloudy skies, where an open-circuit voltage of approximately 7 V and a maximum current of 2 mA were measured.

However, the thermoelectric modules exhibit limited current output. Specifically, two thermoelectric cube modules connected in series produce approximately 600–700 µA at 17 V (measured at the output of the Meissner oscillator circuit). This current level is insufficient to directly supply the voltage regulator and the averaging summing circuit. To mitigate this limitation, the photovoltaic source provides a continuous and relatively stable power contribution, even under suboptimal illumination conditions.

**Figure 26:**
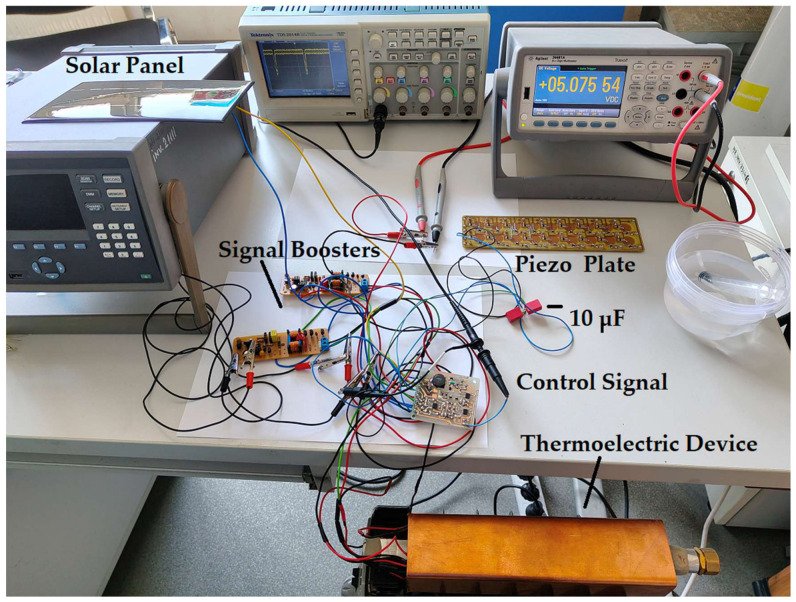
Experimental setup for combining multiple low-power energy sources—solar, thermoelectric, and piezoelectric generators—with complementary characteristics, used to investigate the control signal and the +5 V output of PWM voltage regulator during supercapacitor charging.

Due to their inherently low current output, the thermoelectric and piezoelectric sources alone are unable to sustain a stable input voltage; the voltage rapidly drops below 5 V, causing the voltage regulator to cease operation (see Figure 26). Conversely, the photovoltaic source alone is also insufficient to maintain regulator functionality, due to the 3.5 V reference threshold observed under low-irradiance conditions. This configuration was therefore selected to demonstrate the feasibility and versatility of the averaging summing circuit in integrating multiple low-power energy sources with complementary characteristics.

The regulated output voltage of the converter dropped below 4.8 V only when the input power was insufficient (below 15 mW). For input power levels between 20 mW and 50 mW, the regulator maintained a stable output voltage in the range of 4.9–5.1 V. The output voltage was measured using an Agilent 34461A Digital Multimeter, while control signals were monitored with a Tektronix TDS2014B Oscilloscope.

**Figure 27:**
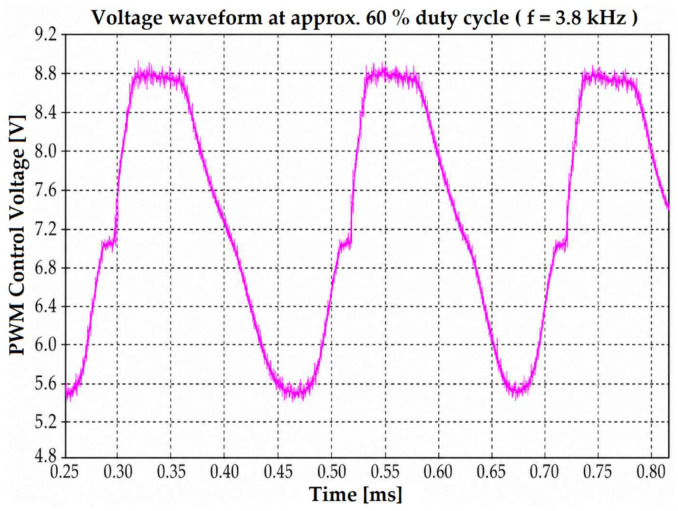
Output waveform of the buck PWM voltage comparator showing a roughly 60% duty cycle at a combined input voltage of 8.8 V.

**Figure 28:**
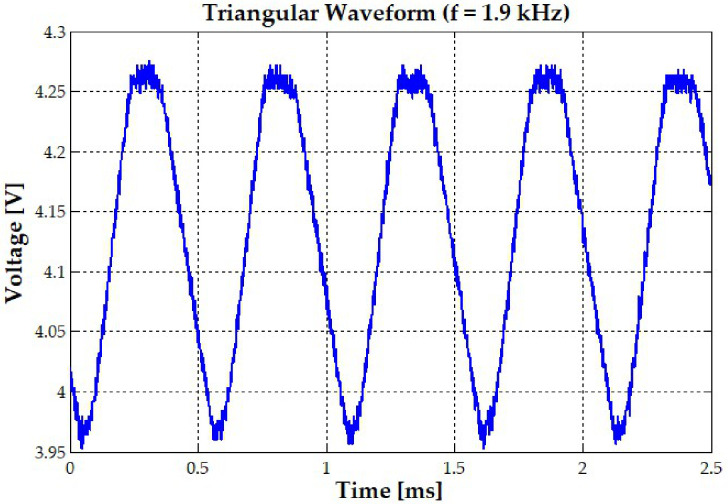
Triangular carrier waveform applied to the non-inverting input of the voltage comparator, centered at 4.12 V.

The ~60% duty-cycle PMOS gate control signal and the triangular carrier waveform were measured using a Tektronix TDS2014B Oscilloscope (see Figure 27 and Figure 28). The triangular waveform was recorded after the duty cycle was established. During this interval, the thermoelectric and piezoelectric sources exhibited a slight decrease in voltage and output power, with the combined input voltage dropping from 8.8 V to 8.2 V.

In PWM-based control, a triangular carrier waveform is compared with the feedback voltage derived from the regulator output to generate a rectangular switching signal, with the duty cycle governed by the input-to-output voltage ratio (for a buck converter duty cycle $D=U_{avg}/U_{out}$). The waveforms from Figure 27 and Figure 28 were acquired under operating conditions in which the thermoelectric voltage boosters produced approximately 10 V each, the piezoelectric bending plate delivered about 12 V after the 20 µF buffer capacitor, and the solar panel generated approximately 7 V under low illumination. Based on (12), the resulting combined voltage at the regulator input was 8.8 V. The triangular carrier waveform operated in the range of 1.9–2 kHz, while the comparator output signal used for gate drive exhibited a frequency of approximately 3.8–4 kHz. This relationship is consistent with the expected behavior of comparator-based PWM generation, where two switching events occur per carrier period. The output feedback voltage was maintained around 4.15 V, positioning it well within the carrier amplitude range (3.95–4.27 V), thereby ensuring a stable duty cycle (approximately 60–65%) and reliable switching operation with adequate noise margin.

Traditional buck converter architectures often struggle with the trade-off between sensing complexity and dynamic performance. To address this, recent advancements propose novel control schemes employing dual-loop mechanisms that decouple switching frequency from output regulation [38,39]. For instance, by utilizing a constant switch-ON time alongside a variable switch-OFF time modulated by output voltage feedback, these systems achieve superior transient response and faster stabilization.

The OP293 integrated circuit can be replaced with the TSV912 in applications where enhanced dynamic performance is required. This substitution enables a significantly improved transient response, primarily due to its higher slew rate (increasing from 0.3–1 V/µs to 5–8 V/µs) and the ability to operate at higher frequencies in the range of 50–100 kHz. However, this performance gain comes at the expense of increased power consumption, with the supply current rising to approximately 0.8–1 mA per amplifier, representing the principal design trade-off in the design decision.

## 4. Discussion

In this work, two energy-harvesting solutions intended for low-power autonomous applications were investigated: a flexible composite piezoelectric device and a compact thermoelectric device based on cube-type modules mounted on an aluminum tube. The results confirm that both structures are capable of converting ambient mechanical or thermal energy into usable electrical output, while also illustrating the importance of geometry, excitation conditions, and thermal management for performance optimization.

For the piezoelectric system, the results showed that the harvested electrical output is governed by the strain distribution along the composite plate, as well as by the number and arrangement of the piezoelectric elements. The central area of the plate was identified as the most effective energy-generating region because it is subjected to the highest bending strain. At the same time, the proposed distributed arrangement allows the structure to exploit the strain energy developed over the entire plate, rather than only in a localized active zone, which improves the overall effectiveness of mechanical-to-electrical energy conversion. The use of separate rectification for each piezoelectric element ensured efficient charge collection and prevented cancellation effects associated with direct parallel coupling. Experimentally, the 18-element structure provided the highest performance, reaching approximately 62 V, 84 µA, and a maximum harvested power of about 5.2 mW, while the 9-element structure delivered about 2.2 mW under comparable excitation conditions. An additional advantage of the proposed concept is related to fabrication: the piezoelectric elements were not adhesively bonded to the composite substrate, but mechanically fixed in position by means of four pop rivets and spacers. This constructive solution simplifies the assembly process, improves replaceability and positioning flexibility, and may be particularly beneficial for large-area or industrial-scale manufacturing of piezoelectric composite plates. Consequently, the proposed design combines good electrical performance with practical advantages in terms of modularity, robustness, manufacturing feasibility, and efficient utilization of the strain energy available in the whole composite plate.

Thin supercapacitors and electrolytic capacitors act as energy buffers, ensuring stable power delivery and minimizing energy losses associated with the intermittent nature of piezoelectric energy harvesting systems [40]. However, additional energy losses arise in the rectification stage due to the forward voltage drop across the bridge rectifier diodes. Although bridge rectifiers based on conventional diodes offer a simple and reliable solution, their inherent voltage drop results in power dissipation, which becomes particularly significant in low-voltage energy harvesting applications. A common approach to reducing these losses is the use of Schottky diodes, which exhibit a lower forward voltage drop than conventional p–n junction diodes. In the present work, BAS40-04 Schottky diode SMD arrays were utilized for each piezoelectric element, providing a forward voltage drop of approximately 0.15–0.20 V per diode at microampere-level currents, thereby reducing rectification losses to 2% in the energy harvesting circuit and preventing charge cancellation between elements [41]. Nevertheless, this approach does not completely eliminate rectification losses. For ultra-low-voltage outputs generated by thin-film piezoelectric harvesters, rectifier diodes can be replaced by bipolar junction transistors (BJTs) or MOSFETs configured as active rectifiers, thereby further improving the conversion efficiency. Such solutions are especially suitable for implementation in integrated circuit designs [40].

For the thermoelectric device, the experiments showed that the proposed cube-module structure mounted on a central aluminum tube can generate useful milliwatt-level power under several operating scenarios. When the copper sheet was additionally heated and cold spring water was introduced into the tube, the generated power reached approximately 10.87 mW at a hot-side temperature of 25 °C, while even at 22.2 °C the power remained around 4 mW. Under dry-tube heating conditions, without water inside the tube, the output still increased from about 1 mW at 24 °C to a maximum of approximately 7.3 mW at 29 °C, which shows that the device can also harvest energy directly from the temperature difference between a warm body or heated surface and the ambient environment. In the passive cooling regime, when only ice-cold water was introduced into the aluminum tube while the copper sheet was initially near ambient temperature, the device generated 2.1–2.6 mW for a short interval, with an average power of about 2.3 mW for nearly 30 s, and still produced about 1.23 mW at a temperature difference of 2 K. The proposed design, which allows the introduction of cold or ice-cold water into the tube, proved effective for creating a temporary thermal gradient, but the results also showed that static water cooling maintains this gradient for a limited duration of 7–12 min.

These results confirm an important practical advantage of the proposed design: the inner cooling pipe can harvest energy not only when an external heat source is applied, but also directly from the temperature difference between the environment and the cold medium stored inside the tube. In addition, the sealed tube configuration, designed according to the principle of a vacuum thermos, helps preserve the cold side for longer periods, while the closed cube cavities may provide supplementary thermal insulation and could be further improved by vacuum filling or phase-change materials. The structure also offers several system-level advantages, including compact integration of four cube-type modules, good thermal homogenization on the hot side through the copper sheet, the possibility of extending operation by forced water circulation or PCM filling, and adaptability to other heat sources such as solar heating or winter soil–air gradients. At the same time, the results showed that the power output tends to saturate as the temperature difference increases, mainly because the internal electrical resistance rises nonlinearly with temperature, which limits the current despite the higher generated voltage.

Taken together, the results highlight the complementary nature of piezoelectric and thermoelectric harvesting. The piezoelectric structure is more suitable for intermittent mechanical excitation [42], while the thermoelectric structure is advantageous when a temporary thermal gradient can be established. Therefore, the integration of both approaches into a hybrid harvesting platform represents a promising direction for self-powered wearable and portable IoT systems.

The results indicate that, under static water conditions, the system reaches a maximum estimated power output of approximately 50 mW. In contrast, when dynamic water movement is introduced, the estimated generated power is at least an order of magnitude higher. This enhancement is attributed to the substantially reduced convective thermal resistance of the water, which falls below 0.1 K/W, thereby enabling more efficient heat transfer and significantly improved energy conversion performance. This comparison highlights the critical role of fluid motion in enhancing heat transfer and, consequently, significantly improving the overall energy harvesting performance.

Future work should focus on improving thermal insulation and forced cooling in the thermoelectric device, optimizing the mechanical flexibility and strain distribution of the piezoelectric plate, and further enhancing the integration between the harvesters and the already implemented power-management circuit to ensure stable and efficient operation in practical applications.

## 5. Conclusions

This work presented a hybrid piezoelectric–thermoelectric energy harvesting system combined with an electronic storage and power-management stage based on supercapacitors. The hybrid energy harvesting system is also supported by a 125 × 195 mm solar panel. Two semi-flexible piezoelectric composite plates, containing nine and 18 PZT elements, were designed and experimentally investigated together with a compact thermoelectric device composed of four cube-type modules arranged around a central aluminum tube. The results demonstrate that both harvesting structures are capable of producing milliwatt-level electrical power under practical mechanical and thermal excitation conditions.

The piezoelectric composite plates showed a strong dependence of the generated electrical output on the applied pulling force, plate geometry, and spatial position of the PZT elements. The configuration with 18 piezoelectric elements provided the highest output, reaching approximately 62 V under dynamic measurement conditions and a maximum harvested power of about 5.2 mW at high mechanical excitation. The 9-element configuration generated a lower but still useful power level of approximately 2.2 mW.

To facilitate comparison with previously reported flexible energy harvesters, the output performance was evaluated in terms of power density. Literature studies typically report power densities in the range of 58.7–286 mW/m^2^ [3]. In the present work, the prototype incorporating 18 piezoelectric elements achieved a power density of 443 mW/m^2^. The semi-flexible plate containing nine PZT elements reached a power density of 328 mW/m^2^. These values exceed the upper range commonly reported in the literature, demonstrating the effectiveness of the proposed design and its potential for enhanced vibration energy harvesting applications.

The use of individual Schottky bridge rectifiers for each piezoelectric element allowed the generated charges to be collected independently and summed on the common output bus, reducing cancellation effects and improving the utilization of the distributed strain field.

The thermoelectric device also demonstrated effective power generation under low-temperature gradients and static water conditions (vacuum thermos configuration). Compared with flexible thermoelectric devices reported in the literature, which typically achieve maximum power densities in the range of 20–30 μW/cm^2^ [8,9], the proposed system demonstrates improved performance. The developed thermoelectric device delivered an output power of 25 mW over a total active copper area of 25 cm × 6 cm × 4 tube faces, corresponding to an effective power density of approximately 42.5 μW/cm^2^. Depending on the operating conditions, the four cube-type modules generated power in the milliwatt range, with higher values obtained when the copper sheet was heated and cold or ice-cold water was introduced into the central aluminum tube. The experimental results confirmed that the generated power is limited not only by the available temperature difference, but also by the nonlinear increase in the internal resistance of the thermoelectric modules. Therefore, maintaining a stable temperature gradient through improved cooling, forced water circulation, phase-change materials, or better thermal insulation remains essential for increasing the useful operating time of the device. These aspects will be addressed in detail in a subsequent study. At this stage, only a preliminary estimation has been performed for forced water cooling under low-flow conditions. For forced convection in water at low velocities and temperatures below 20 °C, with the flow regime assumed to remain laminar (Re ≈ 1000, well below the transition threshold of Re < 2300), the convective thermal resistance is typically in the range of 0.10–0.15 K/W. Under these conditions, an estimated electrical power output of approximately 100 mW is anticipated, representing an increase by a factor of four compared to operation under static (natural convection) water conditions.

As this work represents an initial proof-of-concept thermoelectric prototype, low-cost thermoelectric modules (TEC1-12706) were selected instead of dedicated, higher-efficiency thermoelectric generators. This choice was motivated by cost considerations and component availability, despite the known performance advantages of purpose-built TEG modules under equivalent thermal gradients. Future research will concentrate on dedicated thermoelectric generator designs, dynamic water-flow management, and the integration of phase-change materials to sustain the temperature gradient for longer durations.

A key aspect of this work is the integration of the harvesting devices with dedicated electronic circuits for voltage summing, regulation, protection, and energy storage. The low-voltage thermoelectric outputs were processed using Meissner oscillator circuits, while the piezoelectric, thermoelectric, and photovoltaic contributions were combined through an average voltage summing and source-decoupling circuit. This configuration enabled the complementary use of sources with different voltage, current, and temporal characteristics. The ultra-low-power voltage regulator, implemented with OP293 operational amplifiers, a PWM control stage, a low-on-resistance (0.1 Ω) PMOS transistor, and a Schottky diode with an exceptionally low forward voltage drop (0.1–0.15 V) provided a regulated 5 V output suitable for charging the storage stage. While the average voltage summing circuit provides a simple and effective solution for low-power applications (under 100 mW), its scalability becomes limited as power levels increase. For higher-power systems (over 1 W), a Multi-Input Single-Output (MISO) DC–DC converter topology should be employed. For this topology, power from the various input sources is managed through controlled switching, whereby the sources may be connected during different portions of the switching cycle (time-sharing) or allowed to contribute simultaneously, depending on the converter architecture. This approach enables efficient power sharing and power flow management while maintaining a stable output voltage and accommodating sources with different electrical characteristics [43].

The supercapacitor-based storage system demonstrated the feasibility of accumulating harvested energy from multiple low-power sources. The regulated 5 V output was used to charge a 22 mF supercapacitor together with the output filtering capacitor, reaching its full charge (5 V rated voltage) in approximately 100 s under combined source operation.

A 5.6 V low-leakage Zener diode was introduced as an overvoltage protection element for the 5.5 V-rated Panasonic supercapacitor, improving the reliability of the storage stage during transient operating conditions. These results show that the proposed electronic management circuit can convert intermittent and weak energy-harvesting outputs into a more stable stored energy source.

Overall, the proposed system demonstrates the feasibility of combining piezoelectric, thermoelectric, and photovoltaic energy sources with a compact supercapacitor-based storage and regulation circuit. The hybrid approach is particularly suitable for low-power autonomous devices, wireless sensor nodes, portable electronics, and monitoring systems where environmental mechanical and thermal energy sources are available but individually insufficient for stable operation. Future improvements should focus on reducing mechanical stiffness in the piezoelectric plates, improving the thermal isolation and cooling strategy of the thermoelectric device, optimizing the power-management circuit efficiency, and increasing the long-term stored energy capacity through larger or cascaded supercapacitor modules.

## Figures and Tables

**Table 1:** Strain and bending-moment distribution coefficients of the piezoceramic composite plates.

Plate Bending Coefficients	*k*_0_	*k*_1_	*k*_2_	*k*_3_	*k*_4_
*a* = 0.99	1.00	0.96	0.80	0.60	0.30
*a* = 1.00	1.00	0.96	0.82	0.60	0.30
*a* = 1.16	1.00	0.95	0.79	0.54	0.18

**Table 2:** Linear fitting models for the voltage and current of the piezoceramic composite plates.

Composite Plates	Voltage Coef. *A_p_* (V/N)	Voltage Coef. *B_p_* (V)	Current Coef. *C_p_* (µA/N)	Current Coef. *D_p_* (µA)
9 piezo plate	0.9442	−6.832	1.9241	−8.5998
18 piezo plate	1.4007	−3.4376	2.3025	−29.4893

**Table 3:** Quadratic fitting models for the power of the piezoceramic composite plates.

Composite Piezo Plates	Voltage Coef. A_p_ (mW/N^2^)	Voltage Coef. B_p_ (mW/N)	Current Coef. C_p_ (mW)	Maximum Power $P_{p}\;mW$
9 piezo plate	0.001602	−0.010715	−0.05056	2.24 (41 N)
18 piezo plate	0.00400	−0.09885	0.8096	5.20 (47 N)

## Data Availability

The data presented in this study are available on request from the corresponding author. The data are not publicly available due to institutional policies.
